# Neuron-Like Cells Generated from Human Umbilical Cord Lining-Derived Mesenchymal Stem Cells as a New In Vitro Model for Neuronal Toxicity Screening: Using Magnetite Nanoparticles as an Example

**DOI:** 10.3390/ijms21010271

**Published:** 2019-12-31

**Authors:** Uliana De Simone, Arsenio Spinillo, Francesca Caloni, Laura Gribaldo, Teresa Coccini

**Affiliations:** 1 Laboratory of Clinical & Experimental Toxicology, Toxicology Unit, ICS Maugeri SpA-Benefit Corporation, IRCCS Pavia, Via Maugeri 10, 27100 Pavia, Italy; uliana.desimone@icsmaugeri.it; 2 Department of Obstetrics and Gynecology, Fondazione IRCCS Policlinico San Matteo and University of Pavia, 27100 Pavia, Italy; spinillo@smatteo.pv.it; 3 Dipartimento di Medicina Veterinaria (DIMEVET), Università degli Studi di Milano, 20133 Milano, Italy; francesca.caloni@unimi.it; 4 Chemical Safety and Alternative Methods Unit, Directorate F—Health, Consumers and Reference Materials, Directorate General Joint Research Centre, European Commission, 21027 Ispra, Italy; Laura.GRIBALDO@ec.europa.eu

**Keywords:** Fe_3_O_4_ nanoparticles, environmental toxicology, alternative methods, safety assessment, cell-based assay, toxicity-testing strategies, human primary cell culture, predictive nanotoxicology

## Abstract

The wide employment of iron nanoparticles in environmental and occupational settings underlines their potential to enter the brain. Human cell-based systems are recommended as relevant models to reduce uncertainty and to improve prediction of human toxicity. This study aimed at demonstrating the in vitro differentiation of the human umbilical cord lining-derived-mesenchymal stem cells (hCL-MSCs) into neuron-like cells (hNLCs) and the benefit of using them as an ideal primary cell source of human origin for the neuronal toxicity of Fe_3_O_4_NPs (magnetite-nanoparticles). Neuron-like phenotype was confirmed by: live morphology; Nissl body staining; protein expression of different neuronal-specific markers (immunofluorescent staining), at different maturation stages (i.e., day-3-early and day-8-full differentiated), namely β-tubulin III, MAP-2, enolase (NSE), glial protein, and almost no nestin and SOX-2 expression. Synaptic makers (SYN, GAP43, and PSD95) were also expressed. Fe_3_O_4_NPs determined a concentration- and time-dependent reduction of hNLCs viability (by ATP and the Trypan Blue test). Cell density decreased (20–50%) and apoptotic effects were detected at ≥10 μg/mL in both types of differentiated hNLCs. Three-day-differentiated hNLCs were more susceptible (toxicity appeared early and lasted for up to 48 h) than 8-day-differentiated cells (delayed effects). The study demonstrated that (i) hCL-MSCs easily differentiated into neuronal-like cells; (ii) the hNCLs susceptibility to Fe_3_O_4_NPs; and (iii) human primary cultures of neurons are new in vitro model for NP evaluation.

## 1. Introduction

Among the different types of engineered NPs nanoparticles (NPs), the superparamagnetic iron oxide nanoparticles (SPIONs) are particles formed by small crystals of iron oxide commonly called magnetite Fe_3_O_4_ or maghemite γ-Fe_2_O_3_. These SPIONs have gained a huge interest due to their use for several biomedical and clinical applications (e.g., MRI contrast agents, treatments for anemia, magnetic sensing probes, and drug delivery agent) [1,2].

SPIONs have also been developed for use as environmental catalysts and for incorporation into thermoplastics nanocomposites due to their pigmented properties. The latter include several market products such as car tires, paints, etc. In addition, SPION-based technologies have emerged as promising alternatives to current water and wastewater treatment of organic pollutants as nanoadsorbents or as core component of core-shell structures, where the SPIONs function as magnetic separation and the shell provides the desired functionality for pollutant adsorption [3,4]. Moreover, iron oxide particulates, both fine/micron- and ultra-fine/nano-sized, are generated during anthropogenic activities related to the iron and steel industries, as well as during the NP manufacturing process, where they may represent a significant portion of the circulating air becoming a source of potential hazard for at-risk workers [5].

Therefore, there is a considerable need to address the biocompatibility and safety issues associated with the use of SPIONs.

The promising use of SPIONs for diagnostic and therapeutic applications and their wide utilization in environmental and occupational setting, underline the potential of such SPIONs to enter the brain, making it mandatory to study their potential neurotoxicity [6,7].

A relevant number of studies have demonstrated that engineered nanomaterials (ENMs) could cross the blood–brain barrier (BBB) via different routes, after intentionally and unintentionally exposure and further access the central nervous system (CNS), where ENMs could cause neurotoxicity [8,9,10,11,12,13]. After crossing BBB, ENMs can interact with glial cells and neurons, which could potentially induce a series of disrupted outcomes in the neurological system [14]. The capacity to translocate into the brain has also been demonstrated for SPIONs which, after entering the body, through different routes such as intravenous and intraperitoneal injection, oral administration, and intranasal and intratracheal instillation, gradually accumulate in cerebral tissue, due to their limited excretion, causing damage to neuronal cells and function impairments [15,16]. Several in vivo and in vitro studies have demonstrated the SPIONs neurotoxicity [7,17,18,19,20]. In vivo experimental studies have indicated that Fe_3_O_4_NPs, the predominant form chosen among the SPIONs, can reach the CNS independently of the administration route (e.g., inhalation, intravenous, and intraperitoneal) causing adverse effects in CNS [21,22,23]. In vitro studies have also supported and mechanistically detailed the Fe_3_O_4_NPs-induced neurotoxic effects [24] depending on cell type and surface coating of the NPs [25,26,27,28]. Regarding to the surface coating, different types of natural and synthetic coating materials (such as dextran, pluronic, and polyethylene glycol) and capping agents (such as poly(ethylenimine), and aminosilane) have been used to improve the colloidal stability, interaction, biodistribution, and biocompatibility of SPIONs in biological systems [26,27]. The majority of the in vitro investigations have been performed on different CNS cell types using immortalized cell lines and primary cultures derived from animals, such as PC12, cortical neurons, brain-derived endothelial cells, astrocytes from newborns, microglia (primary or Bv2 cells), and oligodendroglial cells (see review [29]). The major limitation with cell lines is that they are genetically transformed and thus may not represent the normal cell types. Primary cultures have a very limited lifespan and easily lose their tissue-specific characteristics over time. Moreover, it is still difficult to extrapolate animal data to humans because of species differences.

Human cell-based systems are strongly recommended as relevant alternative methods to reduce the uncertainty in species-specific extrapolation of results and to improve prediction in toxicology [30,31]. One of the emerging trends in technologies for developing assays and tools for predictive toxicology goals includes the increased use of stem cells (SCs) [32], which have the advantage, over primary and immortalized cells, of being able to form large populations of stably-differentiated cells representative of different target species including humans. Among SCs, adult stem cells, also known as mesenchymal stem cells (MSCs) are a kind of multipotent progenitors, derived from different human tissues that have a remarkable ability for proliferation, self renewal, and transdifferentiation when they are cultured in specific culture conditions [33,34,35]. Several recent data are reporting their ability to transdifferentiate into the neurogenic lineage [36,37,38,39]. MSCs in vitro differentiation to generate human neuron-like cells (hNLCs) may represent a promising source of cells for neurotoxicity studies.

Among the various sources-derived MSCs, those derived from the human umbilical cord (hUC-MSCs) have the advantages of simple convenient preparation, feasible source, non-traumatic risk of infection, more primitive properties, higher proliferation capacity, and their low immunogenicity and immunosuppressive characteristics turn hUC-MSCs to be an ideal source used as engineering cells in studying stem cell differentiation [40]. Recent investigations have demonstrated that MSCs derived from human umbilical cord can be most efficiently differentiated in vitro into cells of nonmesodermal origin including neuronal-like cells using specific induction protocols [40,41,42,43,44,45]. MSCs can be obtained from different compartments of the umbilical cord (UC) including the UC lining membrane (hCL-MSCs) [45,46,47,48]. These latter cells have been recently isolated and characterized in our lab. The findings have demonstrated that hCL-MSCs may represent a new species-specific tool for establishing efficient platforms for primary screening and toxicity/safety testing of magnetite NPs (Fe_3_O_4_NPs) [49].

According to the development of new strategies for assessing nanomaterial safety, the SC-derived in vitro models may provide more realistic platforms for nontoxicity study [50,51,52], and the differentiation of hMSC into hNLCs can further support their use for screening evaluation of neuronal toxicity of NPs in humans. Indeed current available cell types represent a model less close to the in vivo human neuronal cells, and therefore they are not specifically indicated to characterize neuronal toxicity. This approach reflects the most updated recommendations on using human based models to investigate human diseases and toxic effects from xenobiotics. Moreover significant differences on toxicological profile of Fe_3_O_4_NPs were observed in relation to the cell type model used, rodent versus human [53].

The present study aimed at demonstrating the in vitro differentiation of the human umbilical CL-derived MSCs (hCL-MSCs) into hNLCs (2D-monolayer) and the benefit of using these cells as an ideal primary cell source of human origin for studying the neuronal toxicity of Fe_3_O_4_NPs.

The neuron-like phenotype was confirmed by: (i) live morphological analysis; (ii) Nissl body staining; and (iii) immunofluorescent staining of the expression of different typical neuronal-specific proteins/markers, at different stages of neuron-like cells maturation (i.e., at day 3-early differentiated and day 8-full differentiated), namely β-tubulin III (a microtubule element of the tubulin family, structural marker), MAP-2 (as a mature neuron marker), enolase (NSE, marker characteristic of neural cells), and glial fibrillary acidic protein (GFAP as an astrocyte marker), and almost no expression of nestin (as an immature neuron marker), and SOX-2 (a transcription factor essential for maintaining self-renewal, or pluripotency of undifferentiated stem cells). The synaptic makers such as synaptophysin (SYN, indicator of the synapses density), growth-associated protein 43 (GAP43, a key factor for axonal growth and elongation), and post-synaptic density 95 (PSD95 an important scaffold protein on the post-synaptic membrane, which plays an important role in the process of synapse formation) were also evaluated.

The toxic effects induced by short-term exposure (24–48 h) to increasing Fe_3_O_4_NP concentrations (10–100 μg/mL) have been evaluated on hNLCs of both stages of maturation (i.e., at day 3 and 8). Live/dead cells assessment was pursued applying three assays namely MTT: for identification of metabolic activity (i.e., mitochondrial dehydrogenases); ATP: indicator of ATP production breakdown; and Trypan Blue exclusion test: for membrane integrity determination. Since each of these three assays is using a different endpoint to assess cell viability it cannot be taken for granted that results from these three assays correlate. Moreover and most importantly, since NPs interaction with in vitro assay components and read-out systems may result in a wide array of false positives and false negatives, the suitable in vitro cytotoxicity bioassays need to be verified on case-by-case basis. For studies of potential toxicity of NPs it is recommended to apply at least two different biocompatibility assays that have independent underlying principles, in order to become aware of potential disturbances by NPs on the test systems used [7].

Quantification of apoptosis, by caspase-3/7 activity and nuclear fluorescence staining, and cell morphology analysis by light microscopy were complementary estimated.

Physico-chemical characteristics (i.e., hydrodynamic size, polydispersity Index, Zeta potential, and pH) of Fe_3_O_4_NPs dispersed in the appropriate aqueous media for cell dosing were also assessed, by dynamic light scattering, in order to understand their contribution to toxicity.

## 2. Results

### 2.1. Characterization of Mesenchymal Stem Cells Derived from Umbilical Cord Lining Membrane (Hcl-Mscs): Morphology, Cell Surface Markers, and Differentiation into Adipocytes and Osteocytes

**Figure 1:**
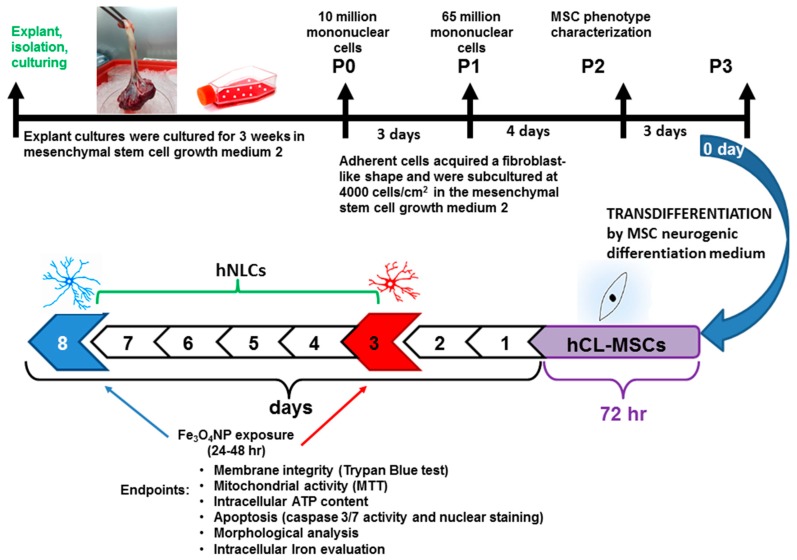
Scheme for the transdifferentiation of hCL-MSCs into neuron-like cells (hNLCs) and the endpoints evaluated at specific timing of Fe_3_O_4_NPs treatment. P indicates the passage number. The appearance of cell outgrowth from explant cultures was routinely monitored, and roundish or long fusiform cells were observed already after 3 days. The typical fibroblast morphology and homogeneous monolayer were detected between the 10th–17th days and the confluence (80–90%) was reached after approximately 21 days from the seeded cord lining (CL) pieces (Figure 1 and Figure 2A).

The set up to obtain and characterize hCL-MSCs and their transdifferentiation into human neuronal-like cells has been performed following the steps reported in Figure 1.

After three weeks, when the cells were transferred to subcultures, their morphology appeared rounded (after digestion) and reverted to the fibroblast-like shape after reattachment to the plate and incubation for 24 h. Thereafter, cell growth was rapid, in about 3–4 days after passage adhered completely to plastic, achieved 80% confluency and showed a uniform spindle-shaped exterior with spiral and radial-like growth.

The cells were cultured until the ninth passage and entered into senescence at the twelfth passage.

**Figure 2:**
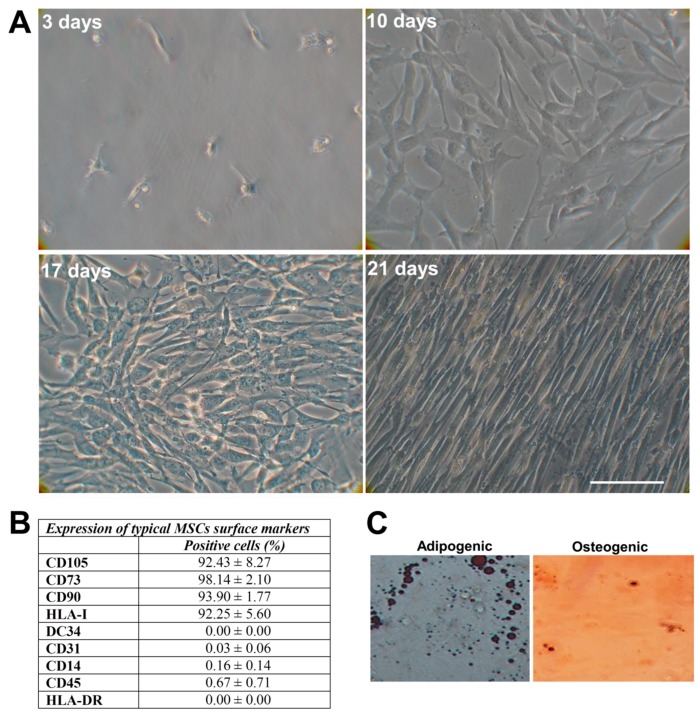
Characterization of mesenchymal stem cells derived from cord lining membrane (hCL-MSCs). (**A**) Morphology of cell outgrowth from the explants of hCL after 3, 10, 17, and 21 days (until P0) as visualized by phase-contrast microscopy (magnification 32×). hCL-MSCs adhere completely to plastic, exhibit fibroblastic morphology and show a radial or spiral growth (clearly visible at 17 days). Scale bar: 100 μm. (**B**) Typical hCL-MSCs surface markers. Percentage mean ± standard deviation (S.D.) (**C**) Representative images of hCL-MSCs after being induced in adipogenic and osteogenic differentiation (magnification 20×).

The immunophenotype was consistent with MSCs: high expression levels of CD73, CD90, and CD105 and HLA-I and negative expression of CD34, CD45, CD14, CD31, and HLA-DR markers (Figure 2B). The hCL-MSCs were capable of differentiating, after the induction in specific media, into adipocytes, as demonstrated by the presence of the intracytoplasmic lipid droplets, and into osteocytes as demonstrated by histological detection of staining for alkaline phosphatase activity and calcium deposition (Figure 2C).

Due to the good growth condition at the 3rd passage cells, these hCL-MSCs were selected for the neurogenic transdifferentiation.

### 2.2. Neurogenic Differentiation

#### 2.2.1. Neuronal-Like Phenotype Characterization

**Figure 3:**
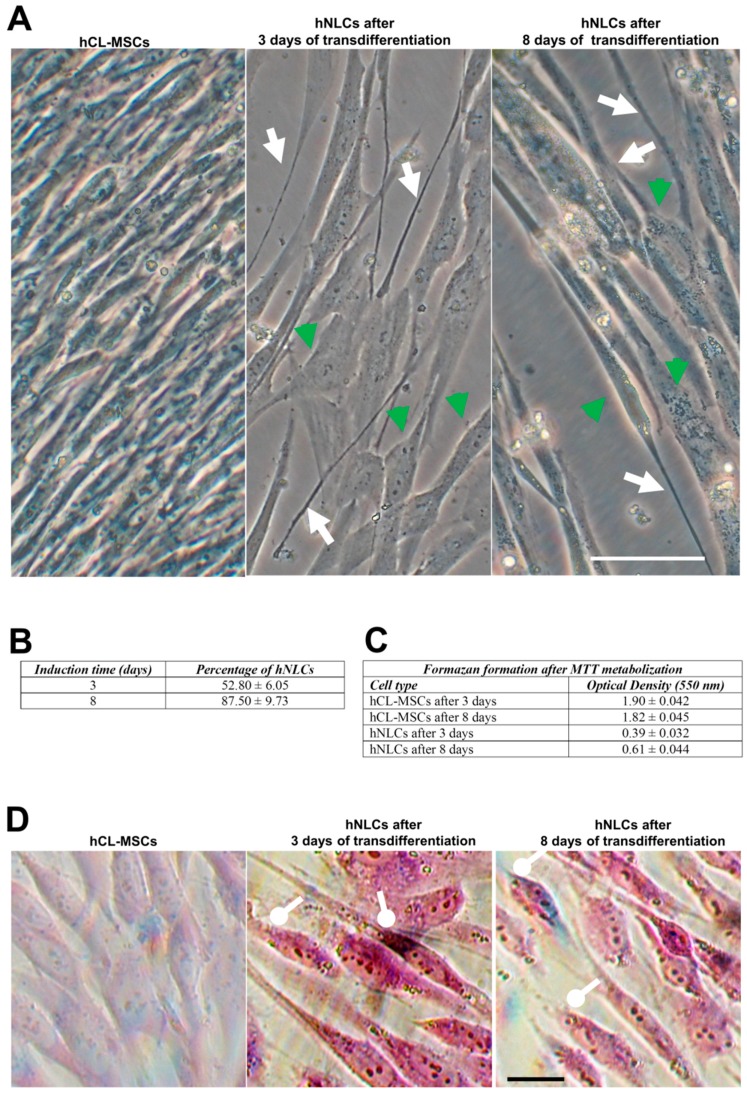
hCL-MSCs transdifferentiated into neuronal lineage at different time points. (**A**) Phase-contrast analysis of hCL-MSCs transdifferentiated into hNLCs at different time points. Cell bodies indicate by green head arrows; elongated structures indicate by white arrows. Scale bar: 100 μm. (**B**) Quantitative changes of hNLCs: the percentage of hNLCs was significantly increased during the induction time. Data are presented as the mean ± S.D. hNLCs at day 3 compared to day 8 of transdifferentiation were statistically different (*p* < 0.05). (**C**) Decrease of cell proliferation capacity during transdifferentiation process into hNLCs (3 and 8 days). Data are presented as the mean ± S.D. (**D**) The Nissl body staining of hCL-MSCs transdifferentiated into neuronal lineage at different time points: differently from the control (hCL-MSCs untransdifferentiated), the hNLCs (after 3 and 8 days) show somata-associated accumulations of the Nissl bodies stained dark black-violet (round-headed white arrows). Scale bar: 100 μm.

**Figure 4:**
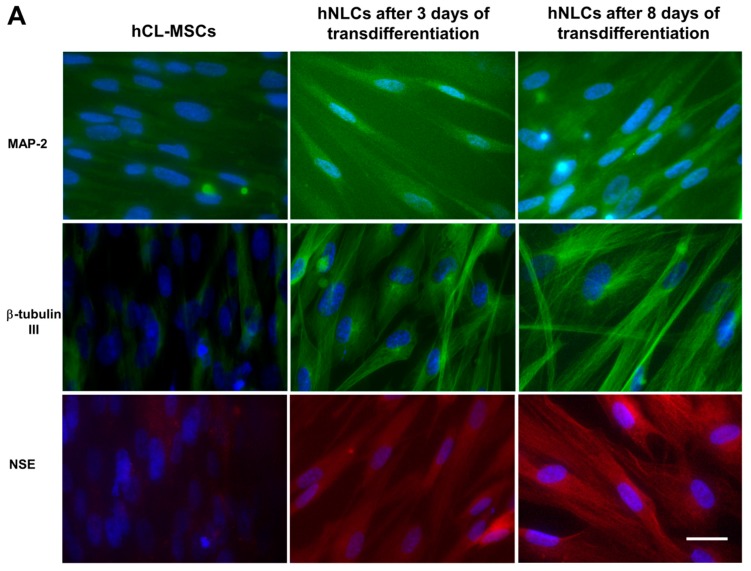

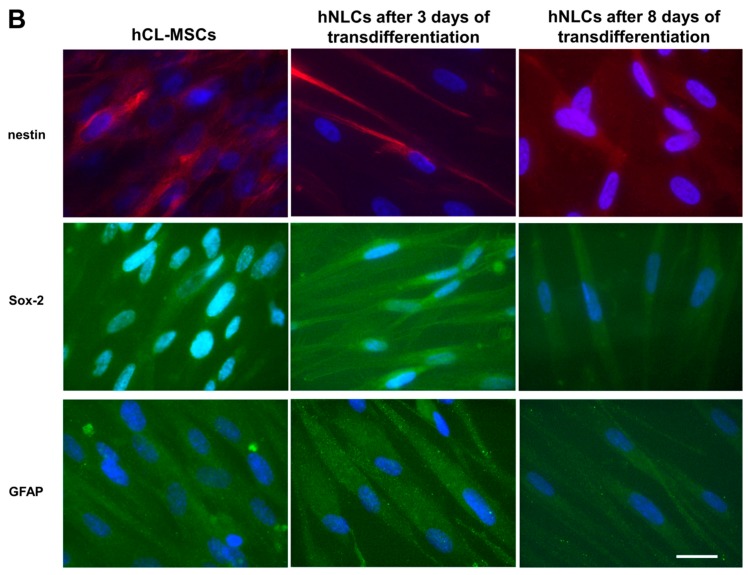
Immunofluorescence characterization of transdifferentiated hNLCs at different time points. (**A**) Representative fluorescence merged microphotographs showing MAP-2- and β-tubulin III-positive (green fluorescence) and enolase-positive (red fluorescence) in hCL-MSCs and transdifferentiated hNLCs at day 3 and 8, (**B**) microphotographs showing nestin-positive (red fluorescence), SOX-2-, and GFAP-positive (green fluorescence) in hCL-MSCs and transdifferentiated hNLCs at day 3 and 8. Nuclei were stained with Hoechst 33258. Scale bar: 100 μm.

**Figure 5:**
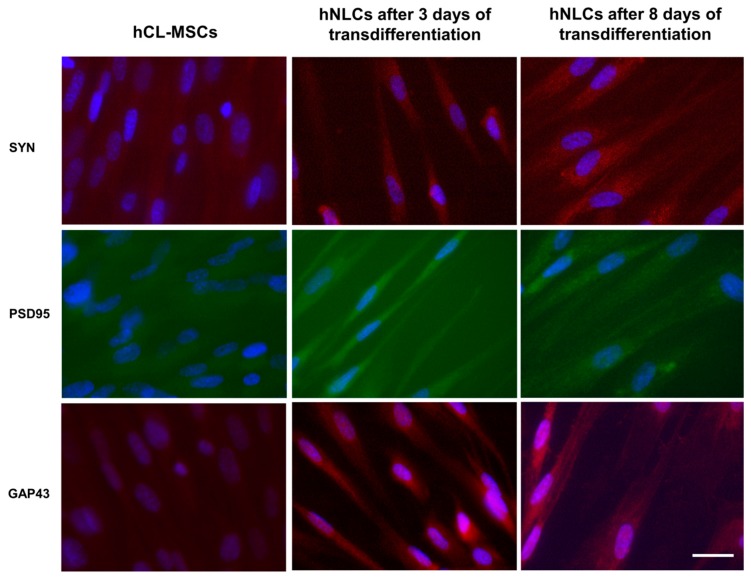
Immunofluorescence of synaptic markers. Representative fluorescence merged microphotographs showing SYN (red fluorescence), PSD95 (green fluorescence), and GAP43 (red fluorescence) positive in hCL-MSCs and transdifferentiated hNLCs at day 3 and 8. Nuclei were stained with Hoechst 33258. Scale bar: 100 μm.

Before assessing the effects of Fe_3_O_4_NPs on neuron-like cells (hNLCs) derived from hCL-MSC transdifferentiation, the neuron-like phenotype was confirmed by: (i) live morphological analysis (Figure 3A), (ii) quantitative changes of hNLCs during differentiation time (Figure 3B) and decrease of cell proliferation capacity (Figure 3C), (iii) Nissl body staining (Figure 3D), and (iv) expression of neuronal and synaptic specific proteins/markers (Figure 4A,B and Figure 5).

##### Morphological and Quantitative Changes of hNLCs at Different Time Points (3 and 8 Days)

The images acquired using contrast-phase microscopy showed that hCL-MSCs transdifferentiated towards a neuronal lineage when cultured in mesenchymal stem cell neurogenic differentiation medium: in fact these induced cells exhibited typical neuron-like morphology (Figure 3A).

On day 3 of transdifferentiation, the cells became oval or round with elongated and extended processes (neurite-like); and the total number of cells that changes versus a phenotype neuron-like reached 52.8% ± 6.05% (Figure 3B). The hNLCs appeared more developed on day 8 of transdifferentiation exhibiting a more advanced neuronal appearance: the length of protrusions increased and gradually intertwine connected into an organized network with adjacent cells (Figure 3A); and about 87.50% ± 9.73% appeared as hNLCs (Figure 3B). On the contrary, the hCL-MSCs cultured in mesenchymal stem cell growth medium 2 showed typical spindle-shape morphology with no changes into neuronal morphology (Figure 3A).

The cell proliferative capacity, evaluated by optical density using formazan formation after MTT metabolization, decreased during the transdifferentiation process into hNLCs (3 and 8 days). The cell density was substantially higher in hCL-MSCs even though the same amount of cells (4000 cells/cm^2^) was seeded for each group (Figure 3C).

##### Nissl Body Staining

The cresyl violet staining labeled the Nissl bodies (granular structures of rough endoplasmic reticulum) in the hCL-MSCs undergoing neurogenic transdifferentiation (hNLCs at 3 days and 8 days of transdifferentiation). The Nissl bodies appeared as dark black-violet spot around the nuclei, while, the same were completely absent in hCL-MSCs cultured in classical mesenchymal stem cell growth medium 2 (Figure 3D).

##### Expression of Neuronal and Synaptic Specific Proteins

The neuronal markers namely MAP-2, β-tubulin III, enolase-NSE, nestin, SOX-2, glial protein-GFAP, and the synaptic makers namely SYN, PSD95, and GAP43, were evaluated after 3 and 8 days of the neurogenic transdifferentiation.

Nuclei were detected using Hoechst 33258 nucleic acid stain, which is a popular nuclear counterstain that emits blue fluorescence when bound to dsDNA.

Figure 4A shows the expression of neuronal markers: MAP-2 and β-tubulin III were visible as green fluorescence around the soma and neurite-like processes in hNLCs at both time points of the neurogenic transdifferentiation, and NSE was visualized as red fluorescent signal into cytoplasm. On the other hand, the MAP-2, β-tubulin III, and NSE antibodies interacted very few with undifferentiated hCL-MSCs. Noteworthy, an improvement of fluorescence intensity of all three of these neuron markers were observed on 8-day hNLCs compared to the 3-day cells (Figure 4A).

Regarding nestin, an early differentiation marker localized in the cytoskeleton, an increase of red fluorescence intensity was observed on 3-day hNLCs, followed by a later decrease of the fluorescence signal after 8 days of transdifferentiation (Figure 4B). In particular, nestin was visible in the vast majority of the hCL-MSCs indicating that these cells were neural stem/progenitor cells (Figure 4B).

By contrast, a weak labeling of SOX-2 (nuclear localization of the fluorescent signal) was detected in both hNLCs when compared to hCL-MSCs: the fluorescence signal decreased during the time of transdifferentiation of these hCL-MSCs into hNLCs (Figure 4B).

Results related to immunofluorescence of GFAP showed the green fluorescence signal in hCL-MSCs and an increase of the fluorescence in hNLCs at 3 days, while hNLCs at 8 days displayed a weak signal, confirming that hCL-MSCs were capable to differentiate not only into hNLCs but also into astrocytes (Figure 4B).

The fluorescence of synaptic makers indicated that the hNLCs (at 3 and 8 days) were able to express both the pre- and post-synaptic proteins such as SYN, PSD95, and GAP43 (Figure 5). Specifically, the anti-SYN staining was exhibited as bright red fluorescence, PSD-95-positive (green fluorescence) was found in proximity to the cell surface, localized to the cell membrane, and GAP43-positive red fluorescence was found in the cell bodies and neurites of hNLCs. Notably, an increase of fluorescence signal for all tested markers were detected on hNLCs at 8 days compared to hNLCs at 3 days and the immunofluorescence was undetectable in hCL-MSC cultures for all markers (Figure 5).

Altogether these findings clearly indicated that hCL-MSCs successfully transdifferentiated into hNLCs. Then this novel in vitro model was used to evaluate the effects induced by short term Fe_3_O_4_NPs exposure on human hNLCs.

### 2.3. Physico-Chemical Characterization of Fe_3_O_4_ Nanoparticles in Mesenchymal Stem Cell Neurogenic Differentiation Medium

The physico-chemical properties of the Fe_3_O_4_NPs suspension (at 10 and 25 μg/mL) in culture medium used for transdifferentiation were investigated in terms of hydrodynamic size, polydispersity index (pdI), zeta potential (Zp), and pH, since they are parameters that affect the outcomes of cellular uptake, distribution, and reactivity of the nanoparticles in the biological system [26].

**Figure 6:**
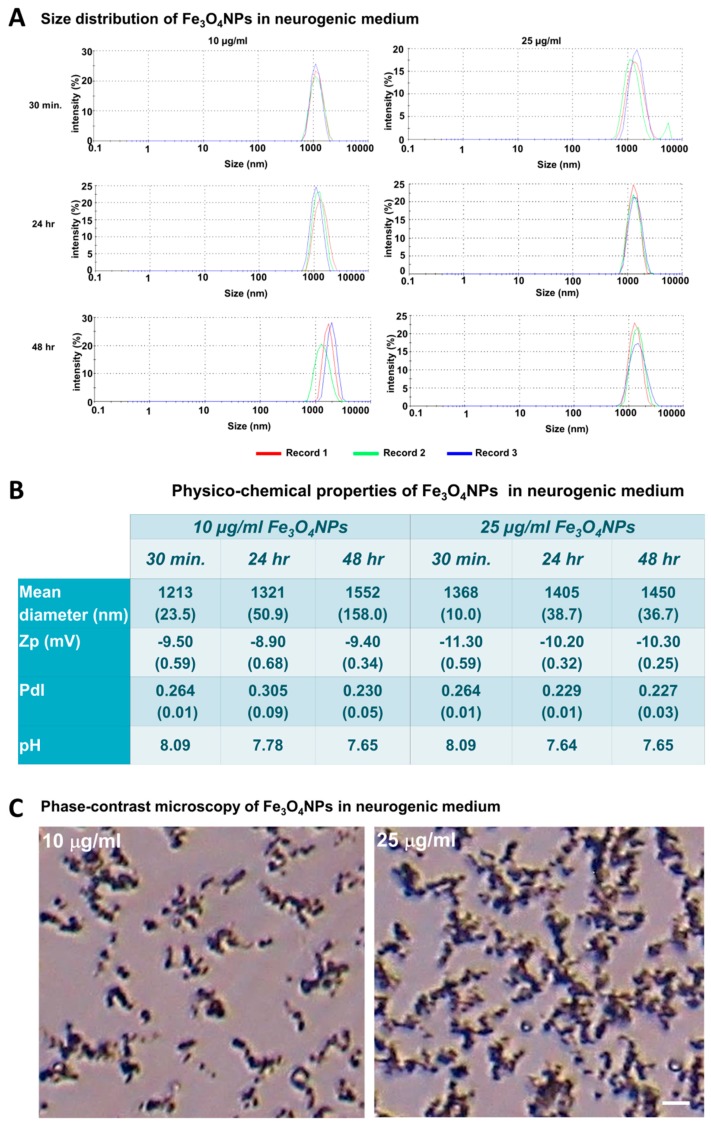
Physico-chemical characteristics of the Fe_3_O_4_NPs in mesenchymal stem cell neurogenic differentiation medium. (**A**) Size distribution obtained from dynamic light scattering measurements of Fe_3_O_4_NPs at concentrations of 10 and 25 μg/mL in mesenchymal stem cell neurogenic differentiation medium after 30 min, 24 and 48 h. (**B**) Physico-chemical properties of the Fe_3_O_4_NPs in mesenchymal stem cell neurogenic differentiation medium. (**C**) Phase-contrast micrographs of Fe_3_O_4_NPs in neurogenic medium at 10 and 25 μg/mL after 48 h: aggregations/agglomerations of the Fe_3_O_4_NPs were observed as brownish sediments, which increased as function of the concentration. Scale bar: 20 μm.

Hydrodynamic size measurements (Figure 6A) showed Fe_3_O_4_NPs agglomeration/aggregation immediately after dispersion in the culture medium (i.e., mesenchymal stem cell neurogenic differentiation medium) exhibiting a size in the micron range: 1213 ± 23.5 and 1368 ± 10 nm at 10 and 25 μg/mL, respectively, after 30 min.

Aggregation still persisted after 24 and 48 h: diameter about 1432 nm as also observed in phase-contrast micrographs that showed aggregation/agglomeration of the Fe_3_O_4_NPs as a function of the concentration (Figure 6B,C).

The pdI values (about 0.266 and 0.240 at 10 and 25 μg/mL, respectively) indicated that the Fe_3_O_4_NPs suspensions were little polydispersed, and also a weak stability in long-term period was evidenced by Zp (around –10 mV) at 30 min, as well as after both 24 and 48 h. The pH values (pH 7.6–8.09) were slightly higher than physiological pH range for each time and concentration point considered (Figure 6B).

### 2.4. Cytotoxic Effects of Fe_3_O_4_NPs Exposure on hNLCs at Different Maturation Stages

Cell viability assays are important tools in toxicology studies to assess the cell sensitivity to compounds. In this study, three widely used cell viability assays, namely MTT (activity of mitochondrial dehydrogenases), Trypan blue (TB; membrane integrity) assays, and ATP (breakdown of ATP production) were applied to assess the Fe_3_O_4_NPs cytotoxicity on neurons. Specifically, hNLCs transdifferentiated at different time points (day 3 and 8) were exposed to increasing Fe_3_O_4_NPs concentrations (10–100 μg/mL) for 24 and 48 h.

#### 2.4.1. MTT Assay

**Figure 7:**
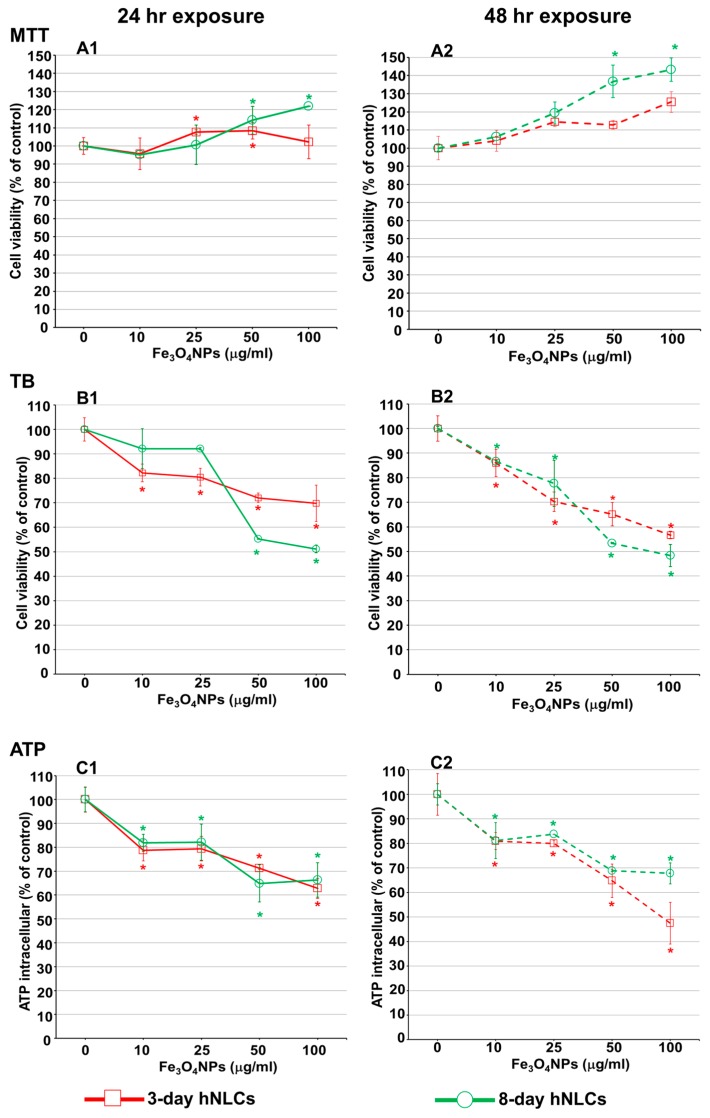
Cytotoxic effects of Fe_3_O_4_NPs on hNLCs treated, at day 3 and 8 of transdifferentiation, for 24 and 48 h with increasing Fe_3_O_4_NPs concentrations (10–100 µg/mL). Evaluation by three cell viability assays. (**A1**,**A2**) Mitochondrial activity assessed by MTT assay; (**B1**,**B2**) cell viability evaluation by Trypan blue (TB) test; and (**C1**,**C2**) evaluation of intracellular ATP content. Data are expressed as percentage of viable cells (% of each control) and represent the mean ± S.D. * *p*< 0.05, statistical analysis by two-way ANOVA followed by Dunnett’s test.

MTT assay, a standard colorimetric method, has long been regarded as the gold standard of cytotoxicity assays as it is highly sensitive. MTT assay is typically applied to evaluate the mitochondrial function by the activity of mitochondrial dehydrogenases after exposure to Fe_3_O_4_NPs. When this test was applied in this study, data indicated an unexpected concentration-dependent increase of cell viability after Fe_3_O_4_NPs treatments in both hNLCs, i.e., early differentiated (at day 3) and fully differentiated stage (at day 8), at both time points considered (24 and 48 h exposure; Figure 7A1,A2).

Cell viability data obtained from MTT assay showed a large difference compared to those obtained from the TB test, ATP assay, and the morphological analysis, which, on the contrary, showed concentration-dependent cell reduction (see below).

The agglomeration/aggregation of Fe_3_O_4_NPs, as evidenced by its physico-chemical characteristics in culture medium (used for transdifferentiation) and its optical properties (supplementary Figure S1) could be factors responsible of interference with the absorbance readings.

#### 2.4.2. Cell Viability Evaluation by a Trypan Blue Exclusion Test

The membrane integrity evaluation on hNLCs was performed using Trypan blue (TB) exclusion test after 24 and 48 h exposure to increasing concentrations of Fe_3_O_4_NPs (10–100 μg/mL) (Figure 7B1,B2).

TB data, on 3-day hNLCs, indicated a gradual reduction of viable cells when compared to control after both time points considered. After 24 h, significant cell viability decrease (about 15%) started at 10 μg/mL with a maximum effect (30% cell viability decrease) at 100 μg/mL (Figure 7B1). The cytotoxicity was exacerbated after 48 h exposure: Fe_3_O_4_NPs treatments induced a significant cell reduction (cell death: 15–45%) at the concentrations ranging from 10 to 100 μg/mL (Figure 7B2).

hNLCs, at day 8-fully differentiated, appeared less susceptible to Fe_3_O_4_NPs than hNLCs at day 3-early differentiated after 24 h exposure: cell viability was affected at the higher concentrations tested, 50–100 µg/mL, with about 50% cell death (Figure 7B1). However, after 48 h, the cytotoxic effects showed similar trend to that observed for hNLCs at day 3-early differentiated (Figure 7B2).

#### 2.4.3. Mitochondrial Metabolism Function by ATP Evaluation

A concentration-dependent reduction of the ATP intracellular content was observed in both hNLCs exposed to 10–100 µg/mL Fe_3_O_4_NPs up to 48 h (Figure 7C1,C2).

Specifically, in 3-day transdifferentiated hNLCs, the ATP intracellular content was reduced from 20% to 35% compared to control following exposure from 10 to 100 μg/mL after 24 h (Figure 7C1) and 20–52% after 48 h (Figure 7C2).

Fe_3_O_4_NP treatments induced similar ATP intracellular depletion (ATP reduction: 15–35% at 10–100 μg/mL) after 24 (Figure 7C1) and 48 h (Figure 7C2) in 8-day transdifferentiated hNLCs.

#### 2.4.4. Evaluation of Caspase-3/7 Activity and Apoptotic Features in Neuron-Like Cells

**Figure 8:**
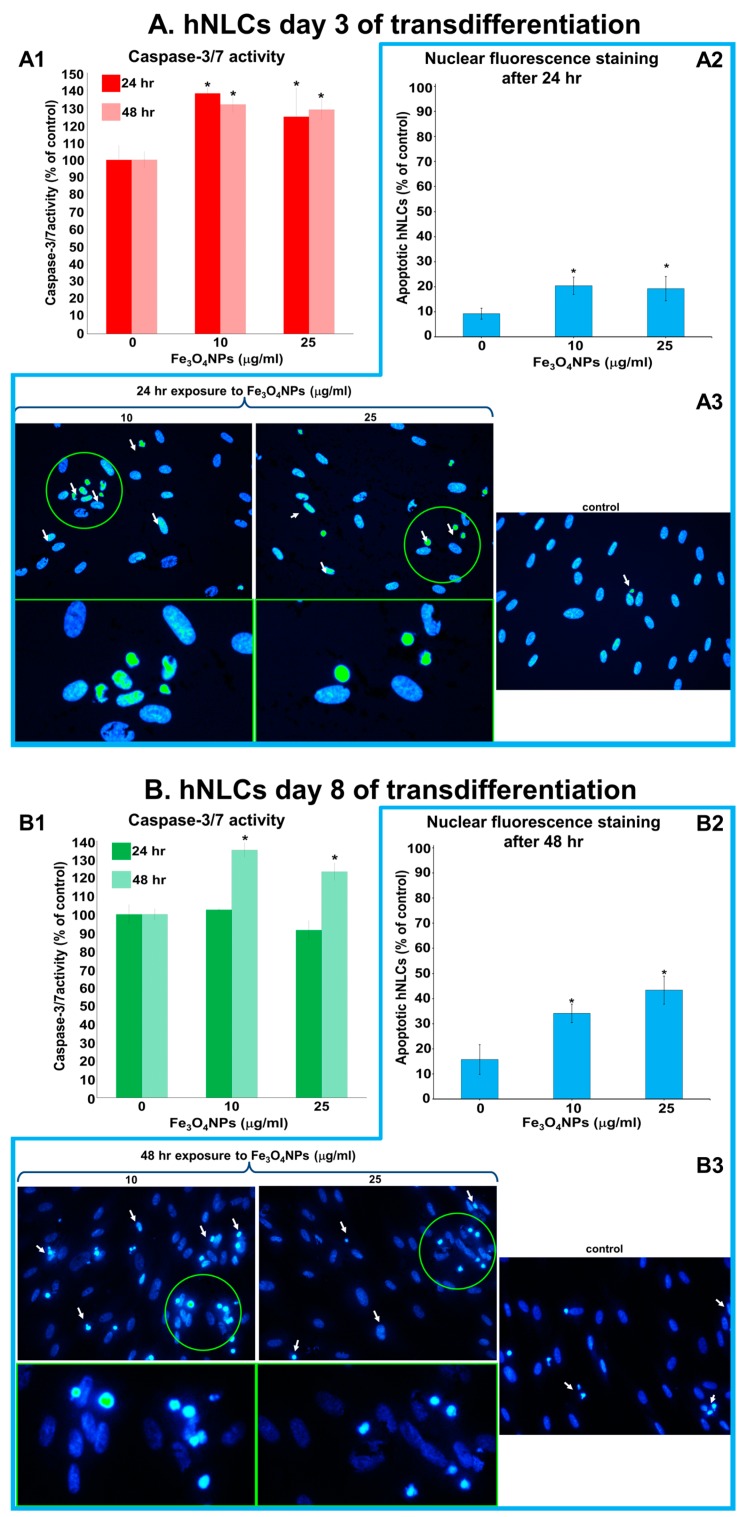
Caspase-3/7 activity and apoptotic cells evaluation after exposure to Fe_3_O_4_NPs (10 and 25 μg/mL). (**A**) Composite images that display the caspase-3/7 activity evaluated after 24 and 48 h exposure to Fe_3_O_4_NPs (**A1**) and apoptotic cells detected by Hoechst 33258 staining after 24 h exposure to Fe_3_O_4_NPs in hNLCs at day 3 of transdifferentiation (**A2**,**A3**). (**B**) Composite images that show the caspase-3/7 activity evaluated after 24 and 48 h exposure to Fe_3_O_4_NPs (**B1**) and apoptotic cells detected by Hoechst 33258 staining after 48 h to Fe_3_O_4_NPs in hNLCs at day 8 of transdifferentiation (**B2**,**B3**). The caspase-3/7 activity of the control cells was set to 100% and the data are expressed as mean ± S.D. * *p* < 0.05. Statistical analysis by two-way ANOVA followed by Dunnett’s test. Apoptotic cells were expressed as % of total cell counted. Data represent the mean ± S.D. * *p* < 0.05. Statistical analysis by two-way ANOVA followed by Dunnett’s test. Representative images in fluorescence microscope are taken using magnification 40×, the areas indicated by the circle show the magnification 2.5-fold. Arrows indicate nuclear morphological changes such as chromatin condensation, fragmentation, and formation of apoptotic bodies.

Caspase-3/7 activity and apoptotic cells were assessed after Fe_3_O_4_NPs treatments in both neuron-like cells (Figure 8A,B). The results obtained on hNLCs after 3 days of transdifferentiation showed that the levels of caspase-3/7 activity increased about 1.33-fold (compared to control) at 10 and 25 µg/mL (Figure 8A1).

The percentage of condensed apoptotic cells was enhanced following treatment with 10 μg/mL and 25 μg/mL of Fe_3_O_4_NPs: about 22% versus 9% detected in control (Figure 8A2). As observed in pictures, the control cells (untreated hNLCs) appeared oval-shaped and the nuclei stained uniformly blue fluorescent (due to the Hoechst 33258 dye). On the contrary, hNLCs (3 days) treated with 10 and 25 μg/mL exhibited typical apoptosis features such as cell shrinkage, chromatin condensation and fragmentation, formation of apoptotic cell bodies, and decrease of cell density (Figure 8A3). The effects lasted for up to 48 h.

In hNLCs (8 days of transdifferentiation) the caspase-3/7 activity was not affected by 24 h treatment with Fe_3_O_4_NPs (Figure 8B1). A significant increase of caspase-3/7 activity, about 1.35-fold (respected to control) at 10 and 25 µg/mL, was instead observed after 48 h exposure, as well as well increase of apoptotic cells percentage (34.15% and 43.36%, at 10 and 25 μg/mL, respectively, compared to control: 15.76%; Figure 6B2). Typical apoptotic morphological changes (including condensation of chromatin and nuclear fragmentation) were observed after 48 h (Figure 8B3).

#### 2.4.5. Morphological Analysis after Fe_3_O_4_NPs Exposure

**Figure 9:**
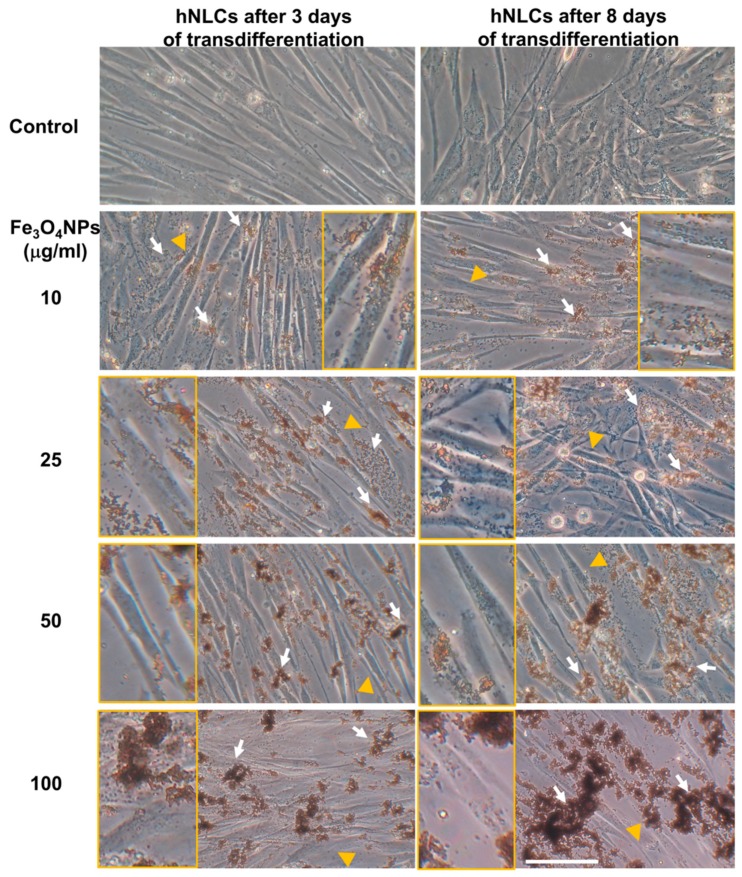
Representative micrographs, by phase-contrast microscopy of early and full transdifferentiated hNLCs after 24 h exposure to increasing Fe_3_O_4_NPs concentrations (10–100 μg/mL). No morphological alterations were observed after Fe_3_O_4_NPs exposure up to 48 h, however, a cell density decrease was observed from 25 μg/mL Fe_3_O_4_NPs at both time points considered for both hNLCs. Brownish aggregates/agglomerates of Fe_3_O_4_NPs in culture medium are indicated by white arrows. Inserts show the magnifications (2×) of the areas indicated by the yellow arrowheads where Fe_3_O_4_NPs are visible inside of the hNLCs. Scale bar: 100 μm.

Cell morphology in both transdifferentiated hNLCs following Fe_3_O_4_NPs exposure was examined by phase-contrast microscopy: no marked morphological changes/alterations have been detected on both hNLCs (early and full differentiated) after Fe_3_O_4_NPs treatments for each time-point considered (Figure 9).

However, cell density decrease was observed from 25 μg/mL Fe_3_O_4_NPs after 24 and 48 h exposure in both neuron-like cell types.

Fine brownish sediments of Fe_3_O_4_NPs were extracellularly visible in both hNLCs at the lowest concentration (10 μg/mL), which became large aggregates/agglomerates at the higher concentrations tested (25–100 μg/mL).

## 3. Discussion

Human MSCs derived from umbilical cord are increasingly under investigation as a promising source for a stem cell-based therapy as well as in vitro model/methods for toxicity screening of drugs/chemicals/nanoparticles, either in studying target organ toxicity or developmental toxicity [52].

The present study supported the evidence that human umbilical CL could be used as an enriched source for the isolation of pluripotent mesenchymal stem cells (hCL-MSCs). In particular, hCL-MSCs are abundantly available and can easily be isolated and expanded from healthy human subjects. These cells met the phenotypical and functional criteria for the definition of MSCs in that they (i) show the typical spindle-shape morphology forming a highly homogeneous monolayer adherent to plastic surfaces; (ii) express adhesion markers CD73, CD105, and CD90 and are devoid of hematopoietic and endothelial markers such as CD31, CD34, and CD45; express positive marker for HLA-I and negative marker for HLA-DR; and (iii) allow adipogenic and osteogenic differentiation in vitro.

Normally, around 65 million of hCL-MSCs are generated at passage 1 from 3 square centimeters of umbilical cord amniotic membrane. Cells grow rapidly, in about 3–4 days after passage adhere completely to plastic, the typical fibroblast morphology and homogeneous monolayer appear between the 10th–17th days, and the confluence (80–90%) is reached approximately 21 days after the seeded CL pieces. The cell cultures with hCL-MSCs require splitting approximately every 3 days from P1 to P9.

All these characteristics are particularly relevant since hUC is considered as a promising source of MSCs due to several advantages compared with other sources of MSCs [48]. Specifically, compared to bone marrow stem cells (the gold standard), hUC-MSCs have a painless collection procedure and faster self-renewal properties. Importantly, the number of mesenchymal stem cells that can be obtained from one UC greatly exceeds the mesenchymal stem cells that can be derived from bone marrow, cord blood, and adipose tissue [46].

By virtue of the inbuilt capacity of stem cells to differentiate, in the present study, the ability of MSCs isolated from human CL to transdifferentiate into hNLCs has been demonstrated applying an easy protocol by using commercially available neurogenic medium, which allow for efficient transdifferentiation observed as phenotypic changes.

The neuronal differentiation process was divided into two stages: early differentiated stage (at day 3) and fully differentiated stage (mature, at day 8). According to our results, cells, that have entered the transdifferentiation pathway, have changed their biological and metabolic properties. The neurogenically induced cells presented a reduced proliferation rate. These hNLCs were positive for enolase and Nissl bodies, and exhibited dendrite-like features of long spikes extending into other adjacent cells and lower cell densities especially after 8 days of transdifferentiation. An early increased and later downregulated level of early differentiation marker proteins nestin and SOX-2 further confirmed the differentiation process. While, increases on neuron marker expression, namely MAP-2 and β-tubulin III were observed during the progression of the differentiation process of the hCL-MSCs into hNCLs. These hNLCs were also positive for the pre- and postsynaptic markers SYN, PSD95, and GAP43. However, whether the obtained hNLCs have synapses capable of transmitting information needs further neuro-electrophysiological studies.

Results were clearly indicating that human umbilical CL-MSCs successfully differentiated into neuronal cells, which would be a predictive preclinical screening tool to evaluate neuronal toxicity in humans.

The ability of hMSCs to transdifferentiate into neurogenic lineage has been confirmed earlier by some authors [36,37,38,54,55] and this capacity has started recently to be investigated for hUC derived-MSCs [40,41,42,43,45,47]. In particular, human cord lining stem cells (hCL-MSCs) overcoming the preexisting difficulties inherent to MSCs from the bone marrow, offer not only a realistic, practical, and affordable alternative for tissue repair and regeneration [46], but also a promising cell-based model for in vitro screening and predictive studies for xenobiotic insults with particularly gained traction due to differentiation ability into several cell lineages including their potential towards converting into neural phenotypes [51,52].

After transdifferentiation and characterization of the hNLCs from hCL-MSCs, the cytotoxicity effects caused by short-term exposure to Fe_3_O_4_NPs have been evaluated on this cellular model. The overall effects were the following:

- MTT data (culture medium with/without the neuron-like cells from hCL-MSCs plus Fe_3_O_4_NPs) indicated increments (artifacts) in cell viability after Fe_3_O_4_NPs exposure. These observations did not fit with the morphological analysis (by phase-contrast microscopy) and the TB and ATP data that overall showed cell density decrease at each time point considered.
- When applying the TB test: (a) on full differentiated neurons (day 8), the cytotoxicity effects appeared at high concentrations (50 µg/mL) after 24 h exposure producing about 50% cell death, (b) while on early differentiated neurons (day 3), a significant cell death (about 20%) appeared at the lowest concentration such as 10 µg/mL Fe_3_O_4_NPs after 24 h exposure, indicating a more susceptibility of the immature compared the mature neurons. After 48 h, the cell reduction became more pronounced compared to that observed after 24 h exposure with similar pattern of toxicity for cells at both stages of the differentiation process.
- A concentration- and time-dependent reduction of the ATP intracellular content (20–35%) was also observed in both hNLCs exposed to 10–100 µg/mL Fe_3_O_4_NPs up to 48 h. The early-differentiated neurons appeared more susceptible in that more marked cell death (about 50%) were detected after 48 h exposure at 100 µg/mL.
- Morphological analyses, parallelly, confirmed cell density decreases after 24 and 48 h exposure to Fe_3_O_4_NPs starting at 25 μg/mL in both neuron-like cell types without alterations of cell morphology.
- Apoptosis induced by low concentrations of Fe_3_O_4_NPs (i.e., 10 and 25 µg/mL) was confirmed by the elevation of caspase-3/7 activity and nuclear staining. The effects appeared early (24 h) on 3-day-differentiated hNLCs, and later (at 48 h) on 8-day-differentiated cells, suggesting a more susceptible of the immature hNLCs than the full differentiated cells.
- Fe_3_O_4_NPs aggregate immediately after dispersion in the culture medium (i.e., mesenchymal stem cell neurogenic differentiation medium). The stability of Fe_3_O_4_NPs suspension (tendency to agglomerate in a specific culture medium) as well as Fe_3_O_4_NP optical properties represent factors that limit in vitro result interpretation depending of the applied methodology. As demonstrated in the present study, the MTT data (artifacts) suggest the not applicability of the spectrophotometric assays for hNLC culture conditions, while TB and ATP are accurate methods for determining cell viability after Fe_3_O_4_NPs exposure in this model.

Altogether these data indicated that Fe_3_O_4_NPs determined a concentration- and time-dependent reduction of human neuron-like cell viability. Cell density decrease (20–50%) were observed at the early time point (24 h) and started at ≥10 μg/mL in both types of differentiated hNLCs in association with apoptotic effects. The 3-day differentiated hNLCs were more susceptible (toxicity effects on both ATP content and membrane integrity started at 10 µg/mL already after 24 h exposure, apoptotic effects also appeared early) than the 8-day differentiated cells (effects on membrane integrity started at higher concentrations after 24 h, and apoptosis at 48 h).

This study enhanced the existing stock of knowledge on the impact of metal oxide NPs on human nervous system in particular. The potential harmful effects of Fe_3_O_4_NPs may be due to iron ion release, which may lead to a disruption of normal iron metabolism/homeostasis in the brain, a characteristic hallmark resembling that of several neurodegenerative disorders (e.g., Alzheimer’s and Parkinson’s) [20,56,57,58]. Indeed, it is well known that too much iron can compromise cell viability, as well as the transport and storage of iron [59]. It could be that Fe_3_O_4_NPs have produced iron liberation that exceeded the iron homeostasis capacity. Our preliminary data using cytochemical Perls’ iron staining are indicating the uptake of Fe_3_O_4_NPs in hNLCs with accumulation in a time- and concentration-dependent manner (data not shown).

Our earlier investigations demonstrated Fe_3_O_4_NP-induced toxicity in 2D mono-cultures of human SH-SY5Y neuroblastoma cells, extensively used in vitro model for CNS toxicity evaluation, after short-term exposure to different concentrations (1–100 µg/mL): cytotoxicity occurred after 48 h only with 35–45% mortality from 10 to 100 μg/mL, whereas no effect was observed at the earlier time point (i.e., 24 h) [60]. Comparatively, the hNLCs of human primary cultures, that are applied in this study, have been shown to be more sensitive to Fe_3_O_4_NPs exposure compared to the SH-SY5Y cell line as toxicity effects were observable early (already after 24 h exposure in the mature cells). Furthermore, by applying this novel in vitro model, information related to NP-induced effects on the early differentiated neurons have also been achieved, revealing the more susceptibility of the immature cells compared the mature ones. In vitro neurotoxicity of Fe_3_O_4_NPs was already evaluated [53,61,62,63], even if the studies on human cell lines are limited [29], and significant differences in cytotoxicity results were observed comparing rodent versus human cells [53], suggesting the importance of the cell type and the species-specific model used to evaluate Fe_3_O_4_NPs toxicological profile. Notably, the findings of the present study further support the use of these human primary cells especially when considering the results obtained in cerebral cells from laboratory animals. In fact, recent studies evidenced a relative resistance of the rat astrocytes and neurons against short-term SPION exposure [25,64,65,66,67], although the experimental conditions [23,65] and the type of SPIONs used [27,67,68] have been shown to influence the toxicity in terms of reactive oxygen species formation and delayed toxic effects in these types of CNS cells.

Our study evidenced that the critical concentrations of Fe_3_O_4_NPs capable to induce in vitro neuronal cytotoxicity are comparable to those measured in peripheral blood and brain tissue of laboratory animals treated with SPIONs. In particular in ICR mice treated with a single intragastric administration of Fe_3_O_4_NPs (13 mg/mouse), the concentrations of Fe_3_O_4_NPs were 375 and 350 μg/mL in the peripheral blood, and 58 and 37 μg/g in brain, respectively at 3 and 10 days post exposure, indicating a relationship between the blood concentration-time changes and brain distribution of Fe_3_O_4_NPs [69]. Moreover, rat intranasally administered iron oxide nanoparticles have been shown to be preferentially distributed in striatum and hippocampus, causing oxidative damage in striatum [20]. The particles deposited at concentrations of 0.040 μg/g and 0.050 μg/g in striata and in hippocampi, respectively. More recently, an in vivo investigation showed that rabbit treated with Fe_3_O_4_NPs exhibited the mitochondrial disease and dysfunction with elevated oxidative stress in the brain [18], and another study provides the direct evidence that locally administered SPIONs in the striatum and hippocampus of mice were able to induce both apoptosis and deficits in some behavioral performance [19].

In humans, elevated iron levels seem to be associated with many types of neurodegenerative disease, such as Alzheimer’s, Parkinson’s, and Huntington’s diseases [70,71,72,73,74]. In this respect, several studies have shown high levels of iron in brain autopsy section from patients with Alzheimer’s disease, Huntington’s disease, and Parkinson’s disease [75,76], leading to the suggestion that elevated iron concentrations within the brain may contribute to the development of neurodegenerative disorders. Moreover, it has also been proposed that some of the excess iron in neurodegenerative tissue may be in the form of the magnetic Fe_3_O_4_ [57]. In support of this hypothesis, concentrations of Fe_3_O_4_ were found to be significantly higher in samples of Alzheimer’s disease tissue [58]. Recently, brain magnetite nanospheres were detected in human subjects and were consistent with an external, rather than an endogenous, source [77]. Their presence proves that externally sourced iron-bearing NPs can be transported directly into the brain, where they can pose hazard to human health.

## 4. Materials and Methods

### 4.1. Chemicals and Reagents

Mesenchymal stem cell growth medium 2 (Ready-to-use; PromoCell, Heidelberg, Germany), mesenchymal stem cell neurogenic differentiation medium (Ready-to-use; PromoCell), human fibronectin solution (1 mg/mL; PromoCell), and all cell culture reagents were purchased from Carlo Erba Reagents (Carlo Erba Reagents S.r.l., Cornaredo, Italy), 75 cm^2^ tissue culture flask with vented filter caps and 96- and 6-well plates (Corning), Trypan blue solution (0.4%), cresyl violet acetate, and MTT ((3-(4,5-dimethylthiazol-2-yl)-2,5-diphenyltetrazolium bromide) were purchased from VWR International PBI (Milan, Italy). CellTiter-Glo^®^ 3D Cell Viability and Caspase-Glo^®^ 3/7 assays were acquired from Promega (Milan, Italy). Primary antibodies (Santa Cruz Biotechnology, Dallas, TX, USA) conjugated to alexa-fluo^®^488 or 594 (Santa Cruz Biotechnology) for nestin, SOX-2, enolase (NSE), MAP-2, β-Tubulin III, synaptophysin (SYN), growth-associated protein 43 (GAP43), post-synaptic density 95 (PSD95), and primary antibody for glial fibrillary acidic protein (GFAP; Santa Cruz Biotechnology) were purchased from D.B.A. Italia s.r.l (Segrate (MI), Italy). Hoechst 33258 (Invitrogen, Waltham, MA, USA) was provided by Life Technologies Italia (Monza, Italy). Polyvinylpyrrolidone coated Fe_3_O_4_NPs were obtained from nanoComposix (San Diego, CA, USA; lot no. ECP1475).

### 4.2. Isolation and Primary Culture of MSCs from Human Umbilical Cord Lining Membrane (hCL-MSCs)

Human umbilical cords (UC, *n* = 9) were obtained from healthy donors, after patients’ provided informed consent (Internal Ethics Committee—Prot. No. 2017000038067, 23 December 2016), who underwent full-term caesarian sections (38–40 weeks’ gestation) at the Hospital Fondazione IRCCS Policlinico San Matteo in Pavia, Italy (from January 2017 to January 2019). In aseptic condition, UC samples were harvested in a collection cup containing physiological solution, store at 4 °C and immediately transported to the lab (within 2 h from collection). The UC samples were washed multiple times with ice-cold phosphate-buffered saline (PBS) to remove blood clots.

From each UC, mesenchymal stem cells (MSCs) derived from human umbilical cord lining membranes (hCL-MSCs) were obtained by the explant method and characterized [49]. Approximately 3 cm in length of UC were collected and the hCL-MSCs were isolated from the subamnion region by dissecting out the Wharton’s jelly followed by removal of cord vessels. The minced pieces of the outer envelope membranes were plated in a 75 cm^2^ tissue culture flask (about 20 tissue pieces/flask) and were maintained in mesenchymal stem cell growth medium 2 at 37 °C, 5% CO_2_ in a humidified atmosphere approximately 3 weeks. In particular CL was minced into 1–2 mm pieces using sterile scissors and CL fragments were incubated in trypsin solution for 30 min at 37 °C for partial digestion, stopped by adding DMEM medium supplemented with 10% heat-inactivated fetal bovine serum (FBS), 2 mM L-glutamine, 50 IU/mL penicillin, and 50 μg/mL streptomycin. About 20 partially digested CL pieces were plated in a 75 cm^2^ tissue culture flask in 10 mL of mesenchymal stem cell growth medium 2. Medium was changed every 3-4 days. Cultures were maintained at 37 °C, 5% CO_2_ in a humidified atmosphere.

The cell growth was monitored every 3–4 days under an inverted phase-contrast microscope. When the mononuclear cells achieved 80–90% confluency, the medium was removed, the cells were rinsed with PBS and then digested with Accutase (up to 5 min at room temperature (r.t.)). Subsequently, the cells were reseeded on 75 cm^2^ flasks at a density of 4000 cells/cm^2^ (P0) and subcultured. Cells split every 3–4 days and cultured up to P4 to be used for the experiments. The remaining cells that were not used for the assay were frozen down at each passage.

Three cm of umbilical cord yielded around 65 million cells at passage 1, in line with literature data [46]. The cells (at passage 2) were characterized for purity and basic MSC characteristics following recommendations of the International Society for Cell Therapy (ISCT) [78,79]: surface markers (CD73, CD34, CD90, CD14, CD45, CD31, CD105, HLA-I, and HLA-DR) analysis and differentiation capacity into adipo- and osteo-lineages (using specific media) were performed as previously described [49]. Briefly, analysis of cell populations was performed with a FACS Navios flow-cytometer (Beckman Coulter) using fluorescein isothiocyanate- or phycoerythrin-conjugated monoclonal antibodies specific for CD73, CD34, CD90, CD14, CD45, CD31, CD105, HLA-I, and HLA-DR. The differentiation capacity of hCL-MSCs was assessed by incubating cells for 21 days with differentiation medium (α-minimal essential medium, 10% fetal bovine serum, 10^−7^ M dexamethasone, 50 mg/mL L-ascorbic acid, and 5 mM β-glycerol phosphate). For adipogenic differentiation 100 mg/mL insulin, 50 mM isobutyl methylxanthine, and 0.5 mM indomethacin were also added. The assessment of adipogenic differentiation was based on the morphological appearance of fat droplets after staining with Oil Red O. To detect osteogenic differentiation, cells were stained for alkaline phosphatase activity using Fast Blue and for calcium deposition with Alizarin Red.

### 4.3. Transdifferentiation of hCL-MSCs into Neuron-Like Cells (hNLCs)

The hCL-MSCs, obtained from *n* = 4 donors, at the 3rd–4th passage according to the logarithmic growth phase, were used for the transdifferentiation into hNLCs and neuronal characterization. The hCL-MSCs were transdifferentiated for 3 days according to the protocol supplied by the manufacturer and up to 8 days to evaluate different stages of maturity (i.e., at day 3-early differentiated and day 8-full differentiated (Figure 1). Specifically, the hCL-MSCs were cultured in standard conditions on 75 cm^2^ tissue culture flask and when about 80% of the cell confluences was reached, the cells were detached by Accutase and reseeded on multiwell plates (at 4000 or 1500 cells per cm^2^) coated with 10 μg/mL human fibronectin, in order to facilitate the cell attachment to the wells, in mesenchymal stem cell growth medium 2.

After 72 h, the cell became subconfluent (about 80%) then the whole culture solution (mesenchymal stem cell growth medium 2) was discarded and replaced with a ready-to-use mesenchymal stem cell neurogenic differentiation medium. Thereafter, the hCL-MSCs were induced in standard growth condition (37 °C/5% CO_2_) and the medium changes were made every 48 h. The cells were cultured for least 3 days up to a maximum of 8 days and they were monitored for the morphological changes using an inverted phase contrast microscope (Zeiss Axiovert 25 microscope equipped with a 32× objective). Parallelly, hCL-MSCs (without passage) were further cultured (for 3 days up to 8 days) in the same condition using growth medium 2 and applied as a control of undifferentiated cells (i.e., hCL-MSCs).

### 4.4. Characterization of the hNLCs

#### 4.4.1. Morphological Changes and Calculation of Positive Rate of hNLCs

On day 3 and 8 of transdifferentiation, the hNLCs were observed under the inverted microscope (Zeiss Axiovert 25 microscope equipped with a 32× contrast phase objective), in order to analyze the morphological changes induced by transdifferentiation. Afterwards, the rate of hNLCs was calculated: three non-overlapping fields were randomly selected and the total cell number and the number of hNLCs were counted.

#### 4.4.2. Immunochemistry Analysis

##### Nissl Body Staining

The transdifferentiation of hCL-MSCs into the hNLCs, on day 3 and 8, were confirmed by the specific staining of neuronal Nissl bodies, characteristic granular structures composed of RNA-rich rough endoplasmic reticulum (rER) unique to the somata of neurons, as follows.

After discarding the culture medium from the hNLCs, they were washed (twice) with phosphate-buffered saline (PBS) prewarmed, and fixed with paraformaldehyde (PF 4%) for 30 min at room temperature (r.t.). Subsequently the hNLCs were washed twice with PBS and stained with Nissl staining solution (0.5% cresyl violet) for 30 min at r.t. After, the staining solution was aspirated and the monolayer was washed three times with PBS, finally mounted with Fluoroshield. The presence of Nissl body into the somata of the hNLCs (3 and 8 days post transdifferentiation) was examined under a CX41 Olympus microscope. Digital images were captured using oil immersion objective (100×) lens.

##### Neuron and Synaptic Markers by Immunofluorescence Analysis

On day 3 and 8 of transdifferentiation, the hNLCs were rinsed with PBS gently and fixed in 4% PF (30 min at r.t.), then permeabilized with 0.1% Triton-100 for 5 min. Subsequently, the hNLCs were washed three times with PBS (containing Ca^2+^ and Mg^2+^) and the samples were incubated with blocking solution (2% dry milk in D-PBS with Ca^2+^ and Mg^2+^) for 30 min at r.t., and then removed without washing followed by incubation with primary antibodies conjugated to alexa-fluo^®^488 or 594 against: nestin (1:100), SOX-2 (2 μg/mL), NSE (1:100), MAP-2 (1:500); β-tubulin III (1:200), SYN (1:100), GAP43 (1:100), and PSD95 (1:100), diluted in 2% dry milk PBS incubated on orbital shaker plate in dark for 60 min at r.t. Next, the monolayers were washed (three times with PBS containing Ca^2+^ and Mg^2+^; 5 min for each washing) and the nuclei were detected using Hoechst 33258 (5 μM for 10 min at r.t.) and finally mounted with Fluoroshield. Only for GFAP, the primary antibody (1:100), was incubated over night at +4 °C, then the cells were washed (three times with PBS containing Ca^2+^ and Mg^2+^; 5 min for each washing), stained with secondary antibody (Alexa 488-labeled; dilution 1:100), washed again with PBS, counterstained for the nuclei and finally mounted with Fluoroshield. For each marker, three separate experiments were performed, each experiments was carried out in three replicates.

Fluorescence images were acquired using a CX41 Olympus fluorescence microscope, excitation light being provided by EPI LED Cassette and combined with digital camera.

Digital images of the eight randomly selected microscopic fields (for each neuronal and synaptic marker) were captured using oil immersion objective (100×) lens, and measurement conditions were the following: 470 nm excitation (T% = 40), 505 nm dichroic beamsplitter, and 510 nm long pass filter.

### 4.5. Fe_3_O_4_NPs Stock Suspension for the Treatment of hNLCs at Different Stages of Maturation

**Figure 10:**
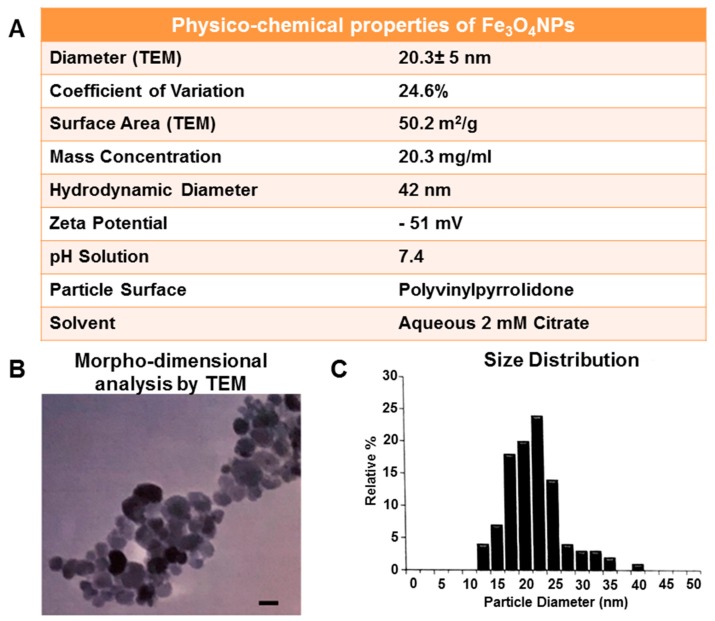
Physico-chemical properties of the Fe_3_O_4_NPs stock suspension in aqueous 2 mM citrate (**A**). Morpho-dimensional analysis of Fe_3_O_4_NPs stock suspension by TEM indicates roughly spherical almost non-agglomerated particles. Scale bar: 20 nm (**B**). Size distribution by dynamic light scattering measurements shows an average diameter of 20.3 ± 5 nm (**C**). Data provided by the Company nanoComposix.

The physico-chemical properties of Fe_3_O_4_NPs stock suspension in a 2 mM citrate solution were provided by the Company (Figure 10).

Briefly, morpho-dimensional analysis using transmission electron microscopy showed that Fe_3_O_4_NPs, dark brown in color, had a roughly spherical, almost non-agglomerated particles, an average diameter of 20.3 ± 5 nm (by transmission electron microscopy) and a hydrodynamic diameter of 42 nm (by dynamic light scattering measurements). The surface of Fe_3_O_4_NPs is functionalized with polyvinylpyrrolidone (PVP), a polymer that offers steric stability and exhibit superparamagnetic properties at ambient temperatures.

Physico-chemical Fe_3_O_4_NPs properties in mesenchymal stem cell neurogenic differentiation medium was also performed: the size of the Fe_3_O_4_NPs and the zeta potential at 10 and 25 µg/mL after 30 min, 24 and 48 h were analyzed by dynamic light scattering using the Malvern Zetasizer Nano ZS90 (N.A.M. S.r.l., NANO-Analysis and Materials, Gazzada Schianno, Varese, Italy).

Fe_3_O_4_NP suspensions were prepared by diluting the stock suspension (20.3 mg/mL) in mesenchymal stem cell neurogenic differentiation medium to obtain increasing Fe_3_O_4_NPs concentrations to be used for neuron-like cells treatments. Fresh solutions of Fe_3_O_4_NPs were prepared and vortexed immediately before each treatment.

### 4.6. Cell Viability Assays

For the Fe_3_O_4_NPs cytotoxicity studies, initially the hCL-MSCs (at P3/4) were seeded in six-well plates at the cell density of 4000 cells/cm^2^ (for the Trypan blue (TB) exclusions test, double staining for apoptosis, morphology) or in 96-well plates at the cell density of 1500 cells/cm^2^ (for the MTT assay, ATP intracellular content, and caspase-3/7 activity), and then following the protocol described in the paragraph “Transdifferentiation of hCL-MSCs into hNLCs”, to obtain the 3-day early and 8-day full differentiated cells.

#### 4.6.1. MTT Assay (Tetrazolium-Based Colorimetric Test Systems Utilizing MTT ((3-(4, 5-Dimethylthiazolyl-2)-2,5-Diphenyltetrazolium Bromide)

The tetrazolium derivatives are reduced in cells mainly by dehydrogenase enzymes, producing intracellular formazan products of specific color that can be measured photometrically after solubilization.

The activity of the dehydrogenase enzymes requires NAD^+^ or NADPH as co-factors, and can therefore be seen as metabolic indicators of cell proliferation. As the result depends on mitochondrial integrity and activity, the sensitivity and general outcome of these assays is dependent on the cellular metabolic activity.

The hCL-MSCs at P3/4 were seeded in 96-well plates (1500 cells per cm^2^) in 100 μL mesenchymal stem cell growth medium 2 per well and incubated (37 °C/5% CO_2_) for 3 days then induced for neuron-like cells transdifferentiation.

Then hCL-MSCs, transdifferentiated into hNLCs, for 3 days up to 8 days, were exposed at both time points to Fe_3_O_4_NPs at concentrations ranging from 10 to 100 μg/mL for 24 and 48 h. After incubation time with Fe_3_O_4_NPs, the cell culture medium was carefully aspirated and hNLCs (early and full differentiated) were washed with PBS (200 μL per well) in order to remove unbound Fe_3_O_4_NPs and to avoid interference with the spectrophotometric analysis. Next, fresh medium (mesenchymal stem cell neurogenic differentiation) plus 10 μL MTT (5 mg/mL) were added to each well and incubated for 3 h. The resulting formazan crystals were solubilized by dimethyl sulfoxide 100 μL per well and quantified by measuring absorbance at 550 nm (measurement) and 655 nm (reference) using a microplate reader (Bio-Rad, Segrate, Milan, Italy). Data were expressed as a percentage of control.

#### 4.6.2. Trypan Blue (TB) Exclusions Test

A loss of integrity of the plasma membrane is seen as one of the hallmarks of necrosis: the uncontrolled and enhanced trans-membrane flow of cytosolic elements or indicator dyes are used as endpoints. One common dye used in toxicity tests is the trypan blue.

On day 3 and 8 of transdifferentiation, the hNLCs were treated with Fe_3_O_4_NPs (ranging from 10 to 100 μg/mL) for 24 and 48 h, untreated control was incubated with mesenchymal stem cell neurogenic differentiation medium only. After each time point, the hNLCs were harvested: Fe_3_O_4_NPs suspensions were removed and cells washed with prewarmed PBS (1 mL/well), detached from the bottom of well by Accutase (500 μL; 5 min. at r.t.), pipetted gently, collected and counted manually using Bürker chamber. TB solution (0.4%) was used in a ratio of 1:10 (viable cells do not take up TB).

#### 4.6.3. ATP Evaluation by CellTiter-Glo^®^ 3D Assay

Another viability-related readout of cellular metabolism is represented by cytosolic ATP content, which can be detected and quantified by luciferin/luciferase assays that generate a luminescence signal proportional to the amount of cytosolic ATP present, which in turn is directly proportional to the number of viable cells present in culture.

On day 3 and 8 of the transdifferentiation, the hNLCs were treated with Fe_3_O_4_NPs (ranging from 10 to 100 μg/mL, for 24 and 48 h) and the ATP content was evaluated using CellTiter-Glo^®^ 3D Cell Viability assay provided as a ready-to-use solution without no additional preparation in according to the protocol supplied by the manufacturer (Promega). Briefly, the CellTiter-Glo^®^ 3D reagent was equilibrated as well as the cells at r.t., for 30 min. Next, the medium was carefully removed and the cells were washed with PBS (200 μL/well) and fresh mesenchymal stem cell neurogenic differentiation medium was added (100 μL/well). Then, 100 μL CellTiter-Glo^®^ 3D reagent were added at each well and the contents were vigorously mixed for 5 min on an orbital shaker plate in order to induce cell lysis. The plate was incubated at r.t. for an additional 25 min (in the dark) to stabilize the luminescent signal. The ATP was quantified by measuring the luminescence signal using a Fluoroskan microplate fluorometer (Thermo Scientific, Milan, Italy) combined with PC software.

The blank reaction (culture medium plus CellTiter-Glo^®^ 3D reagent) was also used in order to measure background luminescence associated with the specific cell culture medium and CellTiter-Glo^®^ 3D reagent. Then, the blank value was subtracted from experimental values.

### 4.7. Assessment of Apoptosis

#### 4.7.1. Caspase-3/7 Activity

Caspase-Glo^®^ 3/7 assay is a luminescence-based test system designed for quantification of caspase-3 and -7 activities. The luminescence signal produced is proportional to the amount of caspase activity present. Caspase-Glo^®^ 3/7 assay is provided as ready-to-use solution without any additional preparation.

On day 3 and 8 of transdifferentiation, the hNLCs were treated with Fe_3_O_4_NPs (ranging from 10 to 100 μg/mL) for 24 and 48 h, then the caspase-3/7 activity was evaluated in according to the protocol supplied by the manufacturer (Promega). Briefly, Caspase-Glo^®^ 3/7 reagent was reconstituted and together with the cells were equilibrated at r.t., for 30 min. Next, the medium was carefully removed and the cells were washed with PBS (200 μL/well) and fresh mesenchymal stem cell neurogenic differentiation medium was added (100 μL/well) as well as the same volume (100 μL/well) of Caspase-Glo^®^ 3/7 reagent. The contents were gently mixed using an orbital shaker plate for 2 h and 30 min at r.t. in the dark. The caspase-3/7 activity was quantified by measuring the luminescence signal using a Fluoroskan microplate fluorometer (Thermo Scientific, Milan, Italy) combined with PC software.

The blank reaction (culture medium plus Caspase-Glo^®^ 3/7 reagent) was also used in order to measure background luminescence associated with the specific cell culture medium and Caspase-Glo^®^ 3/7 reagent. Then, the blank value was subtracted from experimental values.

#### 4.7.2. Nuclear Fluorescence Staining of hNLCs

The hNLCs cell cultures were fixed in PF 4% (20 min at r.t.) and after two washing (PBS) were supravitally stained with the fluorescent nuclear dye, Hoechst 33342 (5 µM for 10 min at r.t.). Afterwards the hNLCs were washed (PBS before then H_2_O), let dry and scored under fluorescence microscope (CX41 Olympus fluorescence microscope equipped with a 40× objective). The microscopic fields were photographed and stored on PC.

### 4.8. Morphology Analysis by Light Phase-Contrast Microscopy

hNLCs, on day 3 and 8 of transdifferentiation, were observed under inverted phase-contrast microscopy after Fe_3_O_4_NPs (10–100 μg/mL) exposure for 24 and 48 h in order to evaluate the healthy status of the cells and their growth. Live-cell microscopy was performed using a Zeiss Axiovert 25 microscope equipped with a 32× contrast phase objective. Images were taken using a digital camera (Canon Powershot G8). Digital photographs were taken and stored on the PC.

### 4.9. Statistical Analysis

Data of the cytotoxicity effects (MTT, ATP, TB, caspase-3/7 activity, and apoptotic cells) were expressed as the mean ± S.D. of three separate experiments, each carried out in four replicates.

Statistical analysis was performed by two-way ANOVA followed by Dunnett’s test. Only *p* values less than 0.05 were considered to be significant.

The intra-assay variation (CV) was <5–8% for the immunophenotyping analysis of hCL-MSCs derived from the same donors. The inter-assay precision ranged between 8% and 10% for the immunophenotyping analysis of the hCL-MSCs derived from different donors.

## 5. Conclusions

The study demonstrated that human umbilical CL-MSCs easily differentiated into neuronal-like cells. These in vitro findings add value to the relevance of using new in vitro human cell-based models in toxicology and, specifically, for the identification of the cytotoxicity of Fe_3_O_4_NPs.

The hCL-MSCs are versatile stem cells easily to be obtained and expanded from healthy human subjects. They have the advantages of strong proliferative ability (self-renewal), long-term proliferation, stable amplification in vitro, wide source without any ethical restriction, and can be easily differentiated into specific cells such as hNLCs. These properties make hCL-MSCs as potential gold standard tool for establishing in vitro models of neurotoxicity and development NT.

With regard to Fe_3_O_4_NPs, the present findings: (i) demonstrate the human neuronal-like cells susceptibility to Fe_3_O_4_NPs exposure, and (ii) support the use of these human primary cultures of neurons as new in vitro species-specific cell model for the evaluation of the NP safety.

The proposed in vitro cell-based model (hNLCs) and the multiple-assays approach, is readily available, either for use as stand-alone method or as a part of integrated strategies, and efficiently valuable to be applied for neurotoxicity testing of nanoparticles.

In addition, this approach is in accordance with the new toxicology paradigm, which emphasizes predictive toxicology (i) more based on in vitro models other than computational systems, bioengineering, automated micro-systems, (ii) boosting basic research more focused to obtain relevant and rapid tools for man, and (iii) understanding the disease cellular mechanisms.

## Supplementary Materials

The following are available online at https://www.mdpi.com/1422-0067/21/1/271/s1, Figure S1: Optical properties of Fe_3_O_4_NPs.

## References

[1] Dulińska-Litewka J., Łazarczyk A., Hałubiec P., Szafrański O., Karnas K., Karewicz A. (2019). Superparamagnetic iron oxide nanoparticles—Current and prospective medical applications. Materials.

[2] Jahangirian H., Kalantari K., Izadiyan Z., Rafiee-Moghaddam R., Shameli K., Webster T.J. (2019). A review of small molecules and drug delivery applications using gold and iron nanoparticles. Int. J. Nanomed..

[3] Gutierrez A.M., Dziubla T.D., Hilt J.Z. (2017). Recent advances on iron oxide magnetic nanoparticles as sorbents of organic pollutants in water and wastewater treatment. Rev. Environ. Health.

[4] Liu X., Zhong Z., Tang Y., Liang B. (2013). Review on the synthesis and applications of Fe_3_O_4_ nanomaterials. J. Nanomater..

[5] Kornberg T.G., Stueckle T.A., Antonini J.A., Rojanasakul Y., Castranova V., Yang Y., Wang L. (2017). Potential toxicity and underlying mechanisms associated with pulmonary exposure to iron oxide nanoparticles: Conflicting literature and unclear risk. Nanomaterials.

[6] Shi D., Mi G., Bhattacharya S., Nayar S., Webster T.J. (2016). Optimizing superparamagnetic iron oxide nanoparticles as drug carriers using an in vitro blood-brain barrier model. Int. J. Nanomed..

[7] Willmann W., Dringen R. (2019). How to study the uptake and toxicity of nanoparticles in cultured brain cells: The dos and don’t forgets. Neurochem. Res..

[8] Hu Y., Gao J. (2010). Potential neurotoxicity of nanoparticles. Int. J. Pharm..

[9] Cupaioli F.A., Zucca F.A., Boraschi D., Zecca L. (2014). Engineered nanoparticles. How brain friendly is this new guest?. Prog. Neurobiol..

[10] Win-Shwe T., Fujimaki H. (2011). Nanoparticles and neurotoxicity. Int. J. Mol. Sci..

[11] Feng X.L., Chen A., Zhang Y., Wang J., Shao L., Wei L. (2015). Central nervous system toxicity of metallic nanoparticles. Int. J. Nanomed..

[12] Bencsik A., Lestaevel P., Canu I.G. (2018). Nano- and neurotoxicology: An emerging discipline. Prog. Neurobiol..

[13] Ge D., Du Q., Ran B., Liu X., Wang X., Ma X., Cheng F., Sun B. (2019). The neurotoxicity induced by engineered nanomaterials. Int. J. Nanomed..

[14] Wang Y., Xiong L., Tang M. (2017). Toxicity of inhaled particulate matter on the central nervous system: Neuroinflammation, neuropsychological effects and neurodegenerative disease. J. Appl. Toxicol..

[15] Song B., Zhang Y., Liu J., Feng X., Zhou T., Shao L. (2016). Is neurotoxicity of metallic nanoparticles the cascades of oxidative stress?. Nanoscale Res. Lett..

[16] Valdiglesias V., Fernandez-Bertolez N., Kilic G., Costa C., Costa S., Fraga S., Bessa M.J., Pasaro E., Teixeira J.P., Laffon B. (2016). Are iron oxide nanoparticles safe? Current knowledge and future perspectives. J. Trace Elem. Med. Biol..

[17] Laffon B., Fernández-Bertólez N., Costa C., Brandão F., Teixeira J.P., Pásaro E., Valdiglesias V., Saquib Q., Faisal M., Al-Khedhairy A.A., Alatar A.A. (2019). Cellular and Molecular Toxicity of Iron Oxide Nanoparticles. Advances in Experimental Medicine and Biology 1048.

[18] Chahinez T., Rachid R., Salim G., Lamia B., Ghozala Z., Nadjiba T., Aya S., Sara H., Hajer C., Samira B. (2016). Toxicity of Fe_3_O_4_ nanoparticles on oxidative stress status, stromal enzymes and mitochondrial respiration and swelling of Oryctolagus cuniculus brain cortex. Toxicol. Environ. Health Sci..

[19] Liu Y., Li J., Xu K., Gu J., Huang L., Zhang L., Liu N., Kong J., Xing M., Zhang L. (2018). Characterization of superparamagnetic iron oxide nanoparticle-induced apoptosis in PC12 cells and mouse hippocampus and striatum. Toxicol. Lett..

[20] Wu J., Ding T., Sun J. (2013). Neurotoxic potential of iron oxide nanoparticles in the rat brain striatum and hippocampus. Neurotoxicology.

[21] Kim Y., Kong S.D., Chen L.H., Pisanic T., Jin S., Shubayev V.I. (2013). In vivo nanoneurotoxicity screening using oxidative stress and neuroinflammation paradigms. Nanomedicine.

[22] Hwang S.R., Kim K. (2014). Nano-enabled delivery systems across the blood-brain barrier. Arch. Pharm. Res..

[23] Petters C., Irrsack E., Koch M., Dringen R. (2014). Uptake and metabolism of iron oxide nanoparticles in brain cells. Neurochem. Res..

[24] Yarjanli Z., Ghaedi K., Esmaeili A., Soheila Rahgozar S., Zarrabi A. (2017). Iron oxide nanoparticles may damage to the neural tissue through iron accumulation, oxidative stress, and protein aggregation. BMC Neurosci..

[25] Petters C., Thiel K., Dringen R. (2016). Lysosomal iron liberation is responsible for the vulnerability of brain microglial cells to iron oxide nanoparticles: Comparison with neurons and astrocytes. Nanotoxicology.

[26] Feng Q., Liu Y., Huang J., Chen K., Huang J., Xiao K. (2018). Uptake, distribution, clearance, and toxicity of iron oxide nanoparticles with different sizes and coatings. Sci. Rep..

[27] Sun Z., Yathindranath V., Worden M., Thliveris J.A., Chu S., Parkinson F.E., Hegmann T., Miller D.W. (2013). Characterization of cellular uptake and toxicity of aminosilane-coated iron oxide nanoparticles with different charges in central nervous system relevant cell culture models. Int. J. Nanomed..

[28] Chen J., Zhu J.M., Cho H.H., Cui K.M., Li F.H., Zhou X.B., Rogers J.T., Wong S.T.C., Huang X.D. (2008). Differential cytotoxicity of metal oxide nanoparticles. J. Exp. Nanosci..

[29] Valdiglesias V., Kiliç G., Costa C., Fernández-Bertólez N., Pásaro E., Teixeira J.P., Laffon B. (2015). Effects of iron oxide nanoparticles: Cytotoxicity, genotoxicity, developmental toxicity, and neurotoxicity. Environ. Mol. Mutagen..

[30] Bal-Price A., Pistollato F., Sachana M., Bopp S.K., Munn S., Worth A. (2018). Strategies to improve the regulatory assessment of developmental neurotoxicity (DNT) using in vitro methods. Toxicol. Appl. Pharmacol..

[31] National Research Council (2007). Toxicity Testing in the 21st Century: A Vision and a Strategy.

[32] Knudsen T.B., Keller D.A., Sander M., Carney E.W., Doerrer N.G., Eaton D.L., Fitzpatrick S.C., Hastings K.L., Mendrick D.L., Tice R.R. (2015). FutureTox II: In vitro data and in silico models for predictive toxicology. Toxicol. Sci..

[33] Fong C.Y., Chak L.L., Biswas A., Tan J.H., Gauthaman K., Chan W.K., Bongso A. (2011). Human Wharton’s jelly stem cells have unique transcriptome profiles compared to human embryonic stem cells and other mesenchymal stem cells. Stem Cell Rev. Rep..

[34] Hsieh J.Y., Fu Y.S., Chang S.J., Tsuang Y.H., Wang H.W. (2010). Functional module analysis reveals differential osteogenic and stemness potentials in human mesenchymal stem cells from bone marrow and Wharton’s jelly of umbilical cord. Stem Cells Dev..

[35] Troyer D.L., Weiss M.L. (2008). Wharton’s jelly-derived cells are a primitive stromal cell population. Stem Cells.

[36] Jang S., Cho H.H., Cho Y.B., Park J.S., Jeong H.S. (2010). Functional neural differentiation of human adipose tissue-derived stem cells using bFGF and forskolin. BMC Cell Biol..

[37] Taran R., Mamidi M.K., Singh G., Dutta S., Parhar I.S., John J.P., Bhonde R., Pal R., Das A.K. (2014). In vitro and in vivo neurogenic potential of mesenchymal stem cells isolated from different sources. J. Biosci..

[38] Zack-Williams S.D., Butler P.E., Kalaskar D.M. (2015). Current progress in use of adipose derived stem cells in peripheral nerve regeneration. World J. Stem Cells.

[39] Buzanska L., Sypecka J., Nerini-Molteni S., Compagnoni A., Hogberg H.T., del Torchio R., Domanska-Janik K., Zimmer J., Coecke S. (2009). A human stem cell-based model for identifying adverse effects of organic and inorganic chemicals on the developing nervous system. Stem Cells.

[40] Shi Y., Nan C., Yan Z., Liu L., Zhou J., Zhao Z., Li D. (2018). Synaptic plasticity of human umbilical cord mesenchymal stem cell differentiating into neuron-like cells in vitro induced by edaravone. Stem Cells Int..

[41] Czarnecka J., Porowińska D., Bajek A., Hołysz M., Roszek K. (2017). Neurogenic differentiation of mesenchymal stem cells induces alterations in extracellular nucleotides metabolism. J. Cell Biochem..

[42] Kil K., Choi M.Y., Park K.H. (2016). In vitro differentiation of human wharton’s jelly-derived mesenchymal stem cells into auditory hair cells and neurons. J. Int. Adv. Otol..

[43] Shahbazi A., Safa M., Alikarami F., Kargozar S., Asadi M.H., Joghataei M.T., Soleimani M. (2016). Rapid induction of neural differentiation in human umbilical cord matrix mesenchymal stem cells by camp-elevating agents. Int. J. Mol. Cell Med..

[44] Singh A.K., Kashyap M.P. (2016). An overview on human umbilical cord blood stem cell-based alternative in vitro models for developmental neurotoxicity. Mol. Neurobiol..

[45] Cortés-Medina L.V., Pasantes-Morales H., Aguilera-Castrejon A., Picones A., Lara-Figueroa C.O., Luis E., Montesinos J.J., Cortés-Morales V.A., De la Rosa Ruiz M.P., Hernández-Estévez E. (2019). Neuronal transdifferentiation potential of human mesenchymal stem cells from neonatal and adult sources by a small molecule cocktail. Stem Cells Int..

[46] Lim I.J., Phan T.T. (2014). Epithelial and mesenchymal stem cells from the umbilical cord lining membrane. Cell Transplant..

[47] Deuse T., Stubbendorff M., Tang-Quan K., Phillips N., Kay M.A., Eiermann T., Phan T.T., Volk H.D., Reichenspurner H., Robbins R.C. (2011). Immunogenicity and immunomodulatory properties of umbilical cord lining mesenchymal stem cells. Cell Transplant..

[48] Ding D.C., Chang Y.H., Shyu W.C., Lin S.Z. (2015). Human umbilical cord mesenchymal stem cells: A new era for stem cell therapy. Cell Transplant..

[49] Coccini T., De Simone U., Roccio M., Croce S., Lenta E., Zecca M., Spinillo A., Avanzini M.A. (2019). In vitro toxicity screening of magnetite nanoparticles by applying mesenchymal stem cells derived from human umbilical cord lining. J. Appl. Toxicol..

[50] Handral H.K., Tong H.J., Islam I., Sriram G., Rosa V., Cao T. (2016). Pluripotent stem cells: An in vitro model for nanotoxicity assessments. J. Appl. Toxicol..

[51] Stueckle T.A., Roberts J.R. (2019). Perspective on current alternatives in nanotoxicology research. Appl. In Vitro Toxicol..

[52] Suma R.N., Mohanan P.V. (2015). Stem cells, a new generation model for predictive nanotoxicological assessment. Curr. Drug Metab..

[53] Ma W., Gehret P.M., Hoff R.E., Kelly L.P., Suh W.H. (2019). The Investigation into the Toxic Potential of Iron Oxide Nanoparticles Utilizing Rat Pheochromocytoma and Human Neural Stem Cells. Nanomaterials.

[54] Woodbury D., Schwarz E.J., Prockop D.J., Black I.B. (2000). Adult rat and human bone marrow stromal cells differentiate into neurons. J. Neurosci. Res..

[55] Martens W., Sanen K., Georgiou M., Struys T., Bronckaers A., Ameloot M., Phillips J., Lambrichts I. (2014). Human dental pulp stem cells can differentiate into Schwann cells and promote and guide neurite outgrowth in an aligned tissue-engineered collagen construct in vitro. FASEB J..

[56] Cengelli F., Maysinger D., Schudi-Monnet F.T., Montet X., Corot C., Petri-Fink A., Hofmann H., Juillerat-Jeanneret L. (2006). Interaction of functionalized superparamagnetic iron oxide nanoparticles with brain structures. J. Pharmacol. Exp. Ther..

[57] Dobson J. (2001). Nanoscale biogenic iron oxides and neurodegenerative disease. FEBS Lett..

[58] Hautot D., Pankhurst Q.A., Khan N., Dobson J. (2003). Preliminary evaluation of nanoscale biogenic magnetite in Alzheimer’s disease brain tissue. Proc. Biol. Sci..

[59] Hohnholt M.C., Dringen R. (2013). Uptake and metabolism of iron and iron oxide nanoparticles in brain astrocytes. Biochem. Soc. Trans..

[60] Coccini T., Caloni F., Ramírez Cando L.J., De Simone U. (2017). Cytotoxicity and proliferative capacity impairment induced on human brain cell cultures after short- and long-term exposure to magnetite nanoparticles. J. Appl. Toxicol..

[61] Au C., Mutkus L., Dobson A., Riffle J., Lalli J., Aschner M. (2007). Effects of nanoparticles on the adhesion and cell viability on astrocytes. Biol. Trace Elem. Res..

[62] Wu J., Sun J. (2011). Investigation on mechanism of growth arrest induced by iron oxide nanoparticles in PC12 cells. J. Nanosci. Nanotechnol..

[63] Wu H.Y., Chung M.C., Wang C.C., Huang C.H., Liang H.J., Jan T.R. (2013). Iron oxide nanoparticles suppress the production of IL-1beta via the secretory lysosomal pathway in murine microglial cells. Part Fibre Toxicol..

[64] Geppert M., Hohnholt M.C., Thiel K., Nürnberger S., Grunwald I., Rezwan K., Dringen R. (2011). Uptake of dimercaptosuccinate-coated magnetic iron oxide nanoparticles by cultured brain astrocytes. Nanotechnology.

[65] Geppert M., Hohnholt M.C., Nürnberger S., Dringen R. (2012). Ferritin upregulation and transient ROS production in cultured brain astrocytes after loading with iron oxide nanoparticles. Acta Biomater..

[66] Geppert M., Petters C., Thiel K., Dringen R. (2013). The presence of serum alters the properties of iron oxide nanoparticles and lowers their accumulation by cultured brain astrocytes. J. Nanopart. Res..

[67] Petters C., Dringen R. (2015). Accumulation of iron oxide nanoparticles by cultured primary neurons. Neurochem. Int..

[68] Rivet C.J., Yuan Y., Borca-Tasciuc D.A., Gilbert R.J. (2012). Altering iron oxide nanoparticle surface properties induce cortical neuron cytotoxicity. Chem. Res. Toxicol..

[69] Wang J., Chen Y., Chen B., Ding J., Xia G., Gao C., Cheng J., Jin N., Zhou Y., Li X. (2010). Pharmacokinetic parameters and tissue distribution of magnetic Fe_3_O_4_ nanoparticles in mice. Int. J. Nanomed..

[70] Beard J.L., Connor J.R., Jones B.C. (1993). Iron in the brain. Nutr. Rev..

[71] Goodman L. (1953). Alzheimer’s disease—A clinicopathologic analysis of 23 cases with a theory on pathogenesis. J. Nerv. Ment. Dis..

[72] Smith M.A., Harris P.L.R., Sayres L.M., Perry G. (1997). Iron accumulation in Alzheimer disease is a source of redox-generated free radicals. Proc. Natl. Acad. Sci. USA.

[73] Thompson K.J., Shoham S., Connor J.R. (2001). Iron and neurodegenerative disorders. Brain Res. Bull..

[74] Zecca L., Youdim M.B., Riederer P., Connor J.R., Crichton R.R. (2004). Iron, brain ageing and neurodegenerative disorders. Nat. Rev. Neurosci..

[75] Collins J.F., Prohaska J.R., Knutson M.D. (2010). Metabolic crossroads of iron and copper. Nutr. Rev..

[76] Zheng W., Monnot A.D. (2012). Regulation of brain iron and copper homeostasis by brain barrier systems: Implication in neurodegenerative diseases. Pharmacol. Ther..

[77] Maher B.A., Ahmed I.A., Karloukovski V., MacLaren D.A., Foulds P.G., Allsop D., Mann D.M., Torres-Jardón R., Calderon-Garciduenas L. (2016). Magnetite pollution nanoparticles in the human brain. Proc. Natl. Acad. Sci. USA.

[78] Bosch J., Houben A.P., Radke T.F., Stapelkamp D., Bünemann E., Balan P., Buchheiser A., Liedtke S., Kögler G. (2012). Distinct differentiation potential of “MSC” derived from cord blood and umbilical cord: Are cord-derived cells true mesenchymal stromal cells?. Stem Cells Dev..

[79] Dominici M., Le Blanc K., Mueller I., Slaper-Cortenbach I., Marini F., Krause D., Deans R., Keating A., Prockop D.J., Horwitz E. (2006). Minimal criteria for defining multipotent mesenchymal stromal cells. The International Society for Cellular Therapy position statement. Cytotherapy.

