# Supplementary material for: Neuron-Like Cells Generated from Human Umbilical Cord Lining-Derived Mesenchymal Stem Cells as a New In Vitro Model for Neuronal Toxicity Screening: Using Magnetite Nanoparticles as an Example
Source: Int J Mol Sci. 2019 Dec 31;21(1):271. doi: 10.3390/ijms21010271 (PMC6982086; doi:10.3390/ijms21010271)
Supplement: Supplementary file 1 [file ijms-21-00271-s001.pdf]

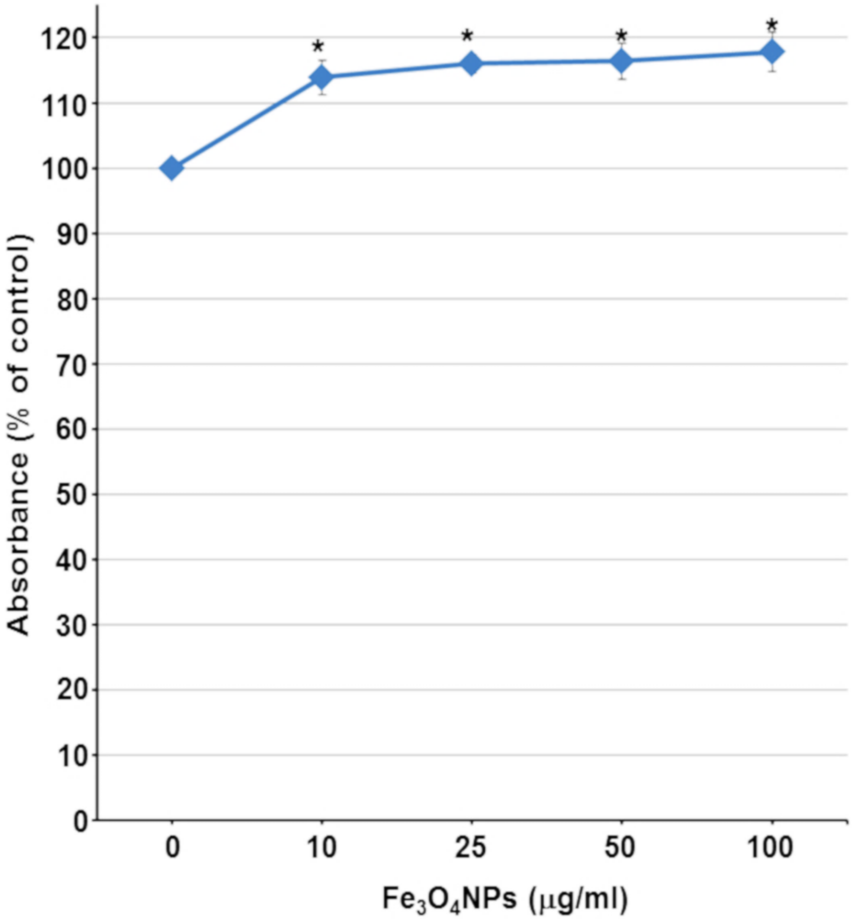

Figure S1. Optical properties of Fe_3O_4NPs alone in mesenchymal stem cell neurogenic differentiation medium evaluated at the same wavelength (λ= 550 nm) of MTT assay. Data are expressed as the absorbance (% of control) and as mean ± S.D. *$p < 0.05$.
